# Fusion genes in pancreatic tumors

**DOI:** 10.1016/j.trecan.2024.01.009

**Published:** 2024-02-19

**Authors:** Anastasios Gkountakos, Aatur D. Singhi, C. Benedikt Westphalen, Aldo Scarpa, Claudio Luchini

**Affiliations:** 1 ARC-Net Research Center, University of Verona, Verona, Italy; 2 Department of Pathology, University of Pittsburgh Medical Center, Pittsburgh, PA, USA; 3 Department of Medicine III and Comprehensive Cancer Centre (CCC), LMU University Hospital Munich and German Cancer Consortium (DKTK), Partner Site Munich, Munich, Germany; 4 Department of Diagnostics and Public Health, Section of Pathology, University and Hospital Trust of Verona, Verona, Italy

## Abstract

Gene fusions and rearrangements play a crucial role in tumor biology. They are rare events typically detected in *KRAS* wild-type (WT) pancreatic tumors. Their identification can inform clinical management by enabling precision oncology, as fusions involving *BRAF*, *FGFR2*, *RET*, *NTRK*, *NRG1*, and *ALK* represent actionable targets in *KRAS*-WT cancers, and serve diagnostic purposes since fusions involving *PRKACA*/*B* represent the diagnostic hallmark of intraductal oncocytic papillary neoplasms (IOPNs). Although they are rare, the therapeutic and diagnostic importance of these genomic events should not be underestimated, highlighting the need for quality-ensured molecular diagnostics in the management of cancer. Herein we review the existing literature on the role of fusion genes in pancreatic tumors and their clinical potential as effective biomarkers and therapeutic targets.

## Gene fusions in solid tumors

In recent years, the advent of novel methodologies and platforms for DNA and RNA sequencing has led to vertical growth in our knowledge of the molecular characteristics of tumors [1]. Recent advances in these techniques have enabled the identification of structural rearrangements in cancer genomes [2,3]. Notably, some of these rearrangements are potential targets for **precision oncology** (see Glossary) [2–4]. Since the initial discovery of the *BCR-ABL* fusion/Philadelphia chromosome in chronic myeloid leukemia [5], an increasing number of gene fusions have been identified in various types of solid tumors, starting with sarcomas [6], and these can serve as effective therapeutic targets. Along these lines, the discovery of **fusion genes** and their roles in cancer biology has greatly contributed to advancing our knowledge of tumorigenesis and has provided opportunities for diagnosis, prognostication, and personalized therapies, as well as **tumor-agnostic treatment**.

Commercially available assays are routinely integrated into clinical practice to support clinical decision-making and personalized treatment approaches for selected patients affected by various types of malignancies, especially in the advanced/metastatic setting. Based on the current knowledge of actionable alterations, these targeted panels can also detect preselected fusion genes of interest [3,4]. However, this targeted approach is not conducive to novel fusion discovery and has inherent limitations stemming from the confines of our current knowledge on this topic [3,4]. Although whole-genome sequencing and whole-transcriptome sequencing (RNA-seq) would be of great help as discovery platforms, even for novel fusions, their clinical application is hindered by their long turnaround times for analysis and high costs [7]. Given these considerations, a critical point to advance precision oncology is the continuous integration of basic research, translational research, and clinical practice.

In the changing scenario of precision oncology, pancreatic cancers represent one of the deadliest solid malignancies and a neoplastic category with few actionable vulnerabilities [8,9]. However, recent investigations, mainly based on individual case series or single case reports, have shown promising results regarding fusion gene-based targeted therapies [10]. Furthermore, other fusion genes detected in pancreatic neoplasms show substantial value in supporting specific diagnoses and/or prognostication.

In this review, we aim to present and analyze the current landscape and potential roles of fusion genes in pancreatic tumors. We discuss the advantages and limitations of the different laboratory tests for the detection of gene fusions along with critical considerations for their implementation in clinical practice. The methodology that has been followed for the systematic review adhered to the PRISMA statement/guidelines [11,12], based on a preset protocol (Table S1 in the supplemental information online); it has been described in detail in Supplementary Methods in the supplemental information online. Moreover, to assess differences in the molecular landscape between cases with and without fusion genes, we used data from **The Cancer Genome Atlas (TCGA)** research network as a reference cohort (Supplementary Methods).

## Presence of fusion genes in pancreatic cancer subtypes

To date, several reports have analyzed the presence of fusion genes in pancreatic tumors [13–73] (Supplementary Methods and Figure S1 in the supplemental information online). Clinicopathological data are summarized in Table 1 and reported in Table S2 in the supplemental information online. Of note, owing to data heterogeneity and differences in the modality of analysis, the real prevalence of fusion genes in pancreatic tumors is difficult to estimate. Analysis of the most important clinicopathological and molecular features of pancreatic tumors harboring fusion genes revealed that such alterations demonstrated a low prevalence, standing at 1.4% in all pancreatic tumors and 1.2% in pancreatic cancer. Globally, 17 044 pancreatic tumors were analyzed for gene fusions, with 2.0% harboring fusion genes. In total, 16 845 pancreatic cancer cases were analyzed, of which 1.6% harbored fusion genes. These percentages (2.0% for pancreatic tumors and 1.6% for pancreatic cancers) may represent a slight overestimation of the real prevalence of this type of genetic alteration in pancreatic tumors/cancers, as some studies have specifically focused only on neoplasms with fusion genes. Thus, to calculate the actual prevalence of fusion genes in pancreatic tumors/cancers, we excluded these studies. Therefore, we estimate the actual prevalence of fusion genes in pancreatic tumors to be 1.4% (242/16 945) and in pancreatic cancers to be 1.2% (208/16 802). Male patients accounted for most cases at 64%, while the mean age of patients was 57.3 years [range 7–86 years, *σ* (standard deviation) = 16.81].

Notably, some specific histotypes appeared to be enriched in fusion genes, including **acinar neoplasms (ANs)**, a category that includes both acinar cell carcinoma and pancreatoblastoma, and intraductal neoplasms compared with **pancreatic ductal adenocarcinoma (PDAC)**. Two hundred and six cases of PDAC and 53 cases of AN harbored fusion genes. The corrected prevalence of fusion events occurred in 1.2% of PDAC cases compared with 19.8% in AN, 100% of IOPNs, and 51% of intraductal tubulopapillary neoplasms (ITPNs). Most molecular analyses were based on an integrated approach, including DNA- and/or RNA-based next-generation sequencing (NGS) and fluorescence *in situ* hybridization (FISH). Simultaneously, a non-negligible proportion of studies were based on a single type of molecular analysis, such as DNA (37.3%) or RNA (10.2%) NGS, or FISH (1.7%).

Although *KRAS* is generally considered the most common and important genetic driver of pancreatic neoplasms, the vast majority (98.0%) of pancreatic tumors harboring fusion genes were *KRAS* WT. Regarding the molecular landscape of pancreatic tumors harboring fusion genes and considering studies reporting NGS data, 58% of cases did not show additional driver alterations. This finding was confirmed when considering only cancers. The most common recurrent genetic alterations in pancreatic tumors with fusion genes involved *TP53* (14.3%), followed by *CDKN2A* and/or *CDKN2B* homozygous deletion (HD) (13.9%) and *SMAD4* mutation/HD (10.9%). In PDAC, recurrent genomic alterations followed a similar distribution of *TP53* mutation (18.6%), *CDKN2A* and/or *CDKN2B* HD (13.2%), and *SMAD4* mutation/HD (9.3%), as well as unique alterations such as *GNAS* mutation (5.4%), *MCL1* amplification (4.7%), and *MYC* amplification (3.9%). Comparing the molecular landscape of PDAC bearing fusion genes, using the findings derived from the current study, with the molecular profile of conventional PDAC without fusion genes, using data from the TCGA cohort, there were significant differences in the prevalence of alterations of classic PDAC drivers. PDAC with fusion genes showed a higher frequency of *KRAS*-WT status and a reduced prevalence of alterations involving *TP53*, *SMAD4*, and *CDKN2A*/*B*. In AN, *CDKN2A* and/or *CDKN2B* HD (27.2%) contained the highest percentage of genomic alterations, followed by *SMAD4* mutations/HD (15.2%) and *TP53* mutations (6.1%).

**Figure 1.**
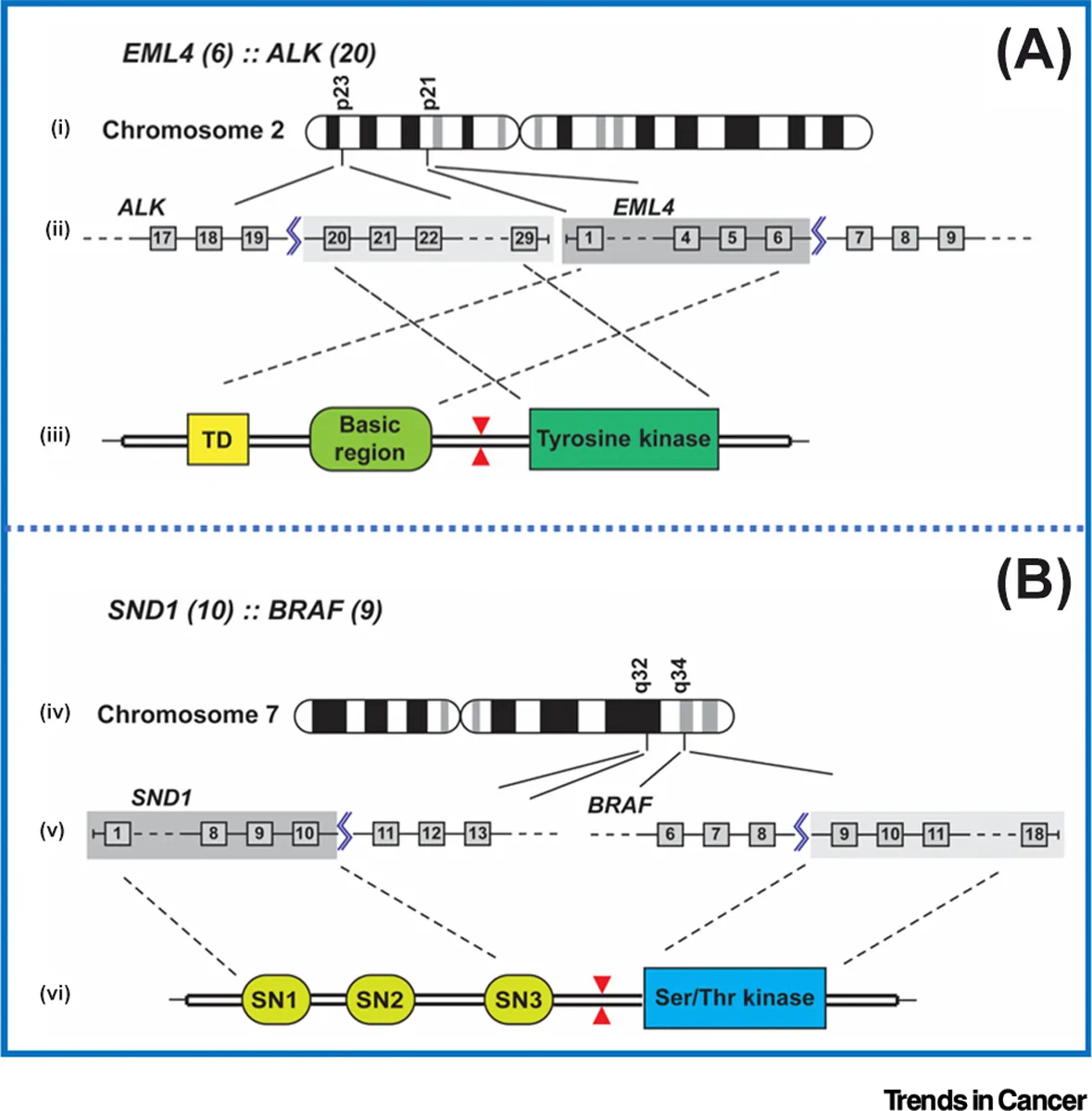
A schematic representation of the two most frequent actionable fusion genes identified in pancreatic cancers: *ALK* (6)::*EML4* (20) and *SND1* (10)::*BRAF* (9), on chromosome 2 and 7, respectively. (A) (i) *ALK* and *EML4* are both located on the short arm (p) of chromosome 2, at 2p21 and 2p23, respectively. (ii) *ALK* contains 29 exons, with the kinase domain mapping from exons 20–29 as a blue symbol indicating the breakpoint. *EML4* contains 28 exons and the breakpoint (blue symbol) creates a part spanning from exon 1 to 6. As a result, exons 1–6 are fused to exons 20–29 creating the *EML4*::*ALK* fusion gene. (iii) The resulting chimeric protein contains the trimerization domain (TD) and basic region originally derived from *EML4* and the tyrosine kinase domain originally belonging to *ALK*. (B) (iv) The *SND* and *BRAF* genes are both located on the long arm (q) of chromosome 7, at 7q32 and 7q34, respectively. (v) *SND1* includes 24 exons and the breakpoint (blue symbol) divides the gene into two parts, with the first containing exons 1–10. *BRAF* contains 18 exons and the breakpoint occurs before exon 9 (blue symbol) with its kinase domain spanning from exon 11 to 18. Exons 1–10 are fused to exons 9–18 forming the *SND1*::*BRAF* fusion gene. (vi) The produced fusion protein comprises three staphylococcal nuclease (SN)-like domains originating from *SND1* and *BRAF*’s serine/threonine kinase domain.

Moreover, when a fusion gene was present, it was the only genetic driver detected in most tumors (58.0%), highlighting its biological importance in tumorigenesis and related mechanisms. Two hundred and seventy-six potentially actionable fusions were detected in pancreatic tumors (Table 2). The most common fusion genes in pancreatic tumors involved *BRAF* (33.7%), *FGFR2* (17.4%), *RET* (12.7%), and *ALK* (10.1%). When both partners of the fusion genes were reported, the most common fusions were *BRAF*::*SND1* and *ALK*::*EML4* (Figure 1). Regarding the specific association between actionable fusion genes and histology, fusions involving *ALK*, *FGFR2*, *NRG1*, *NTRK*, *MET*, and *RET* were typically enriched in PDACs compared with ANs. By contrast, *BRAF* and *RAF* fusions were found across the different histologies and without statistical differences. Interestingly, fusion genes involving *PRKACA*/*B* were detected in all cases of IOPN, emerging as a diagnostic hallmark with statistical significance for this tumor entity.

**Figure 2.**
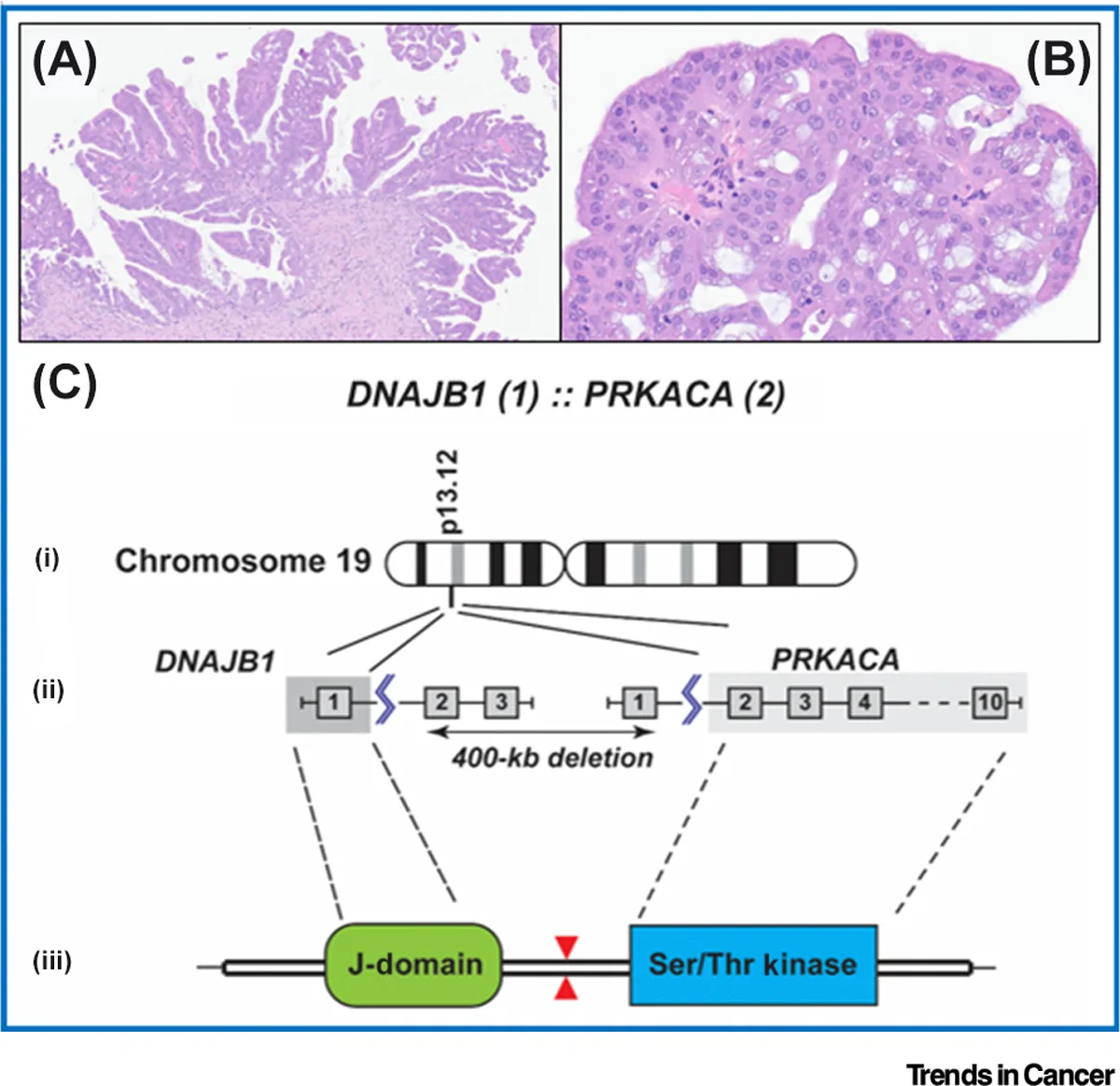
Illustrative figures showing the histomorphology (A,B) and molecular hallmarks (C) of intraductal oncocytic papillary neoplasms of the pancreas. These intraductal lesions show papillary architecture [(A); hematoxylin–eosin, original magnification 4×] and oncocytic cells with high-grade atypia [(B); hematoxylin–eosin, original magnification 40×]. The most common fusion gene is represented by *DNAJB1*::*PRKACA*. (C) (i) *DNAJB1* and *PRKACA* are both located on the short arm (p) of chromosome 19, at 19p13.12. (ii) *DNAJB1* and *PRKACA* contain three and ten exons, respectively. A single 400-kb deletion removes exons 2 and 3 of *DNAJB1* and exon 1 of *PRKACA*, as blue symbols (breakpoints) indicate. As a result, exon 1 is fused to exons 2–10 forming the *DNAJB1*::*PRKACA* fusion. (iii) The chimeric protein contains the J-domain originally derived from *DNAJB1* and the Ser/Thr kinase domain originally belonging to *PRKACA*.

Non-druggable fusion genes are summarized in Table S3 in the supplemental information online. The most non-druggable fusion genes are *PRKACA*/*B* genes, which pair with *ATP1B1* or *DNAJB1*, serving as alternative partners. Such fusions were detected only in IOPNs, reaching statistical significance and suggesting diagnostic potential for this tumor entity (Figure 2).

Based on the low values seen in cases only harboring fusion genes, specific molecular investigations for the detection of only fusion genes in pancreatic tumors are not justifiable, even in the context of precision oncology. However, some specific subcategories may warrant genetic analyses for their detection. ANs, and intraductal neoplasms such as ITPNs and IOPNs, appear to be enriched for fusion genes. Regarding molecular background, all *KRAS*-WT pancreatic tumors, including PDACs, are typically enriched for fusion genes and should be considered for high-throughput sequencing in the context of precision oncology. In this setting it is important to point out that *KRAS*-WT pancreatic cancers typically arise in younger patients.

Overall, fusion genes in pancreatic tumors can play two distinct roles, as potential actionable targets for precision oncology in ANs, invasive cancers derived from ITPNs, and *KRAS*-WT PDAC and as diagnostic hallmarks for IOPNs.

## Precision oncology and related implications

The presence of fusion genes suggests that they could be actionable targets for precision oncology. Existing targeted therapies have been administered to patients with pancreatic neoplasms harboring specific fusion genes. Overall, a total of 59 patients received tailored treatment based on the presence of a fusion gene as the actionable target. Notably, many patients showed a complete (10.2%) or partial (79.6%) response to these regimens, while 6.8% had progressive disease and 3.4% discontinued treatment due to intolerance (Table 3). The most promising results, with a predominance of complete/partial responses to treatment, were observed for fusion proteins involving *FGFR2*, *NTRK*, and *RET*.

Among cases with a complete response, one study described an interesting case of an 80-year-old man with stage IV PDAC harboring a *MET* fusion [37]. After tumor progression to standard chemotherapy (gemcitabine plus nab-paclitaxel), the patient showed a complete response to targeted therapy with crizotinib that is ongoing at >12 months of treatment. Along this line, a case was reported of a 68-year-old woman with stage IV PDAC harboring a *FGFR2* fusion and showing tumor progression to standard chemotherapy [41]. The patient achieved tumor complete response to erdafitinib, a pan-FGFR inhibitor, 2 months after initiation of targeted therapy. Of note, a similar case showed an exceptional response to erdafinitib, in a 28-year-old man with stage IV PDAC harboring a known *FGFR2* fusion [57].

Along this line, one of the largest studies in the context of fusion-based targeted therapy was LIBRETTO-001, a Phase 1/2, single-group, open-label basket trial of selpercatinib in patients with solid tumors harboring *RET* fusions [55]. Among all patients with tumors originating from various organs, the longest period of therapy (45 months at the time of the study’s publication, and still on treatment) was observed in a patient with pancreatic cancer. Of note, selpercatinib received a disease-agnostic approval for RET-positive locally advanced or metastatic solid tumors by the FDA in September 2022 [55,74]. Along with those involving *RET*, other gene fusions with the largest number of patients receiving molecular-based therapy were represented by those involving *ALK*. Notably, the well-established *EML4*::*ALK* fusion is already considered a therapeutic target in non-small cell lung cancer [75] and is one of the most prevalent fusions in PDAC. *ALK* fusions could be considered therapeutic targets in selected patients with pancreatic cancer. These are classic cases to be discussed by molecular tumor boards [7].

Regarding precision oncology, the limited available information highlights the existence of still-open questions, including: (i) the establishment of the optimal timing for treatment of fusion-positive pancreatic cancers; (ii) how to select the preferred molecular target in cases of simultaneous coexistence of fusion genes with another actionable alteration [76] [e.g., a case with *FGFR2* fusion and microsatellite instability (MSI)] [23]; and (iii) the possible role of pancreas-specific cell of origin or tissue microenvironment (desmoplastic stroma, immune cells) in influencing the response to targeted agents against fusion genes. Such considerations highlight the urgent need for further research on fusion genes in pancreatic tumors.

**Figure 3.**
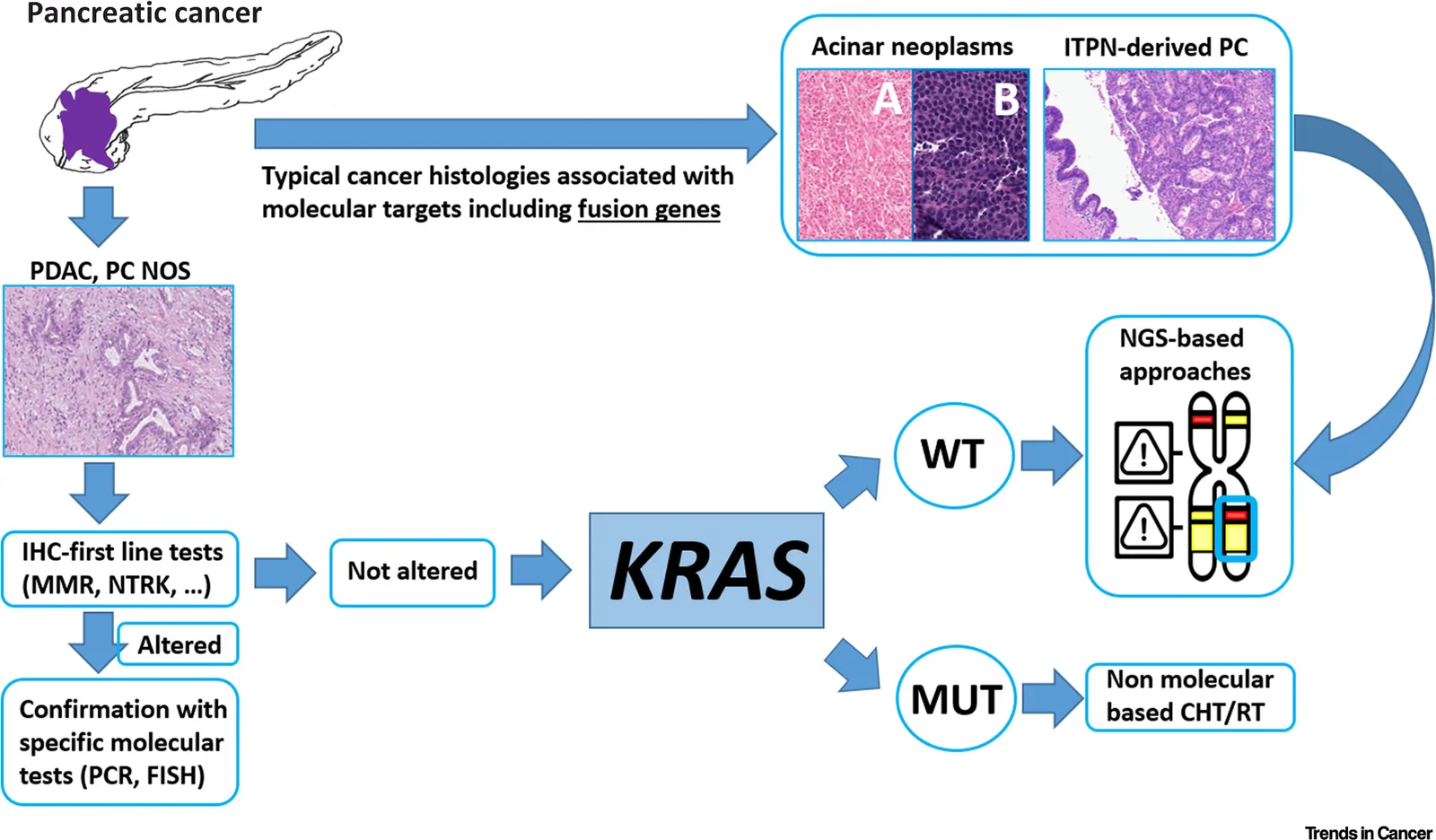
Possible approach to the identification of fusions in pancreatic cancer. Please note that this summary figure has been specifically designed in the context of this review on fusion genes and its main aim is to present a potential approach to the identification of fusions; other important aspects (e.g., status of *BRCA* genes, homologous recombination deficiency) are not considered in this schematic representation. Abbreviations: A, acinar cell carcinoma; B, pancreatoblastoma; CHT, chemotherapy; FISH, fluorescence *in situ* hybridization; IHC, immunohistochemistry; ITPN, intraductal tubulopapillary neoplasm; MMR, mismatch repair protein; MUT, mutated; NGS, next-generation sequencing; PC NOS, pancreatic cancer, not otherwise specified; PDAC, pancreatic ductal adenocarcinoma; RT, radiotherapy; WT, wild type.

Currently, in the context of precision oncology, a possible approach to fusion detection should be based on the integration of histology with molecular pathology (Figure 3). It is important to acknowledge that conventional PDAC lacks histological hallmarks classically associated with the presence of fusion genes. PDAC and its variants and pancreatic cancer, not otherwise specified (PC NOS) could be investigated with immunohistochemistry (IHC) at the time of diagnosis. In this context, IHC can be seen as a universal screening test for the detection of alterations in protein expression with predictive value (e.g., loss of mismatch repair proteins). In the case of alteration, IHC findings should be confirmed by direct molecular tests (e.g., MSI-based PCR for loss of mismatch repair proteins). In those cases lacking IHC alteration, *KRAS* status should be investigated (at least in hotspot regions, with PCR). Based on this analysis, tumors with *KRAS* mutations (MUT) should be treated with conventional approaches of chemotherapy/radiotherapy (CHT/RT), and all *KRAS*-WT pancreatic cancers should be further investigated with NGS. Such tumors are enriched in fusion genes and in other molecular alterations that inform the choice of therapeutic regimens. Of note, pancreatic cancers showing histologies typically associated with the presence of druggable molecular targets including fusion genes, such as AN and carcinomas derived from ITPN, should be directly investigated with NGS. This type of histological–molecular association highlights the important role played by practicing pathologists.

## Detection methods and diagnostics

Critical observations should be made regarding the modality of fusion detection in general and in pancreatic tumors. Various methodologies have been adopted for fusion detection, each with their own advantages and limitations (Table 4). The introduction of new technologies for fusion gene detection into the diagnostic workflow for pancreatic neoplasms is likely to aid in promoting earlier diagnosis and in identifying effective treatment options.

Most investigations adopted an integrated approach with DNA and/or RNA-NGS/FISH. In addition to improving the selection of patients for implementation of gene fusion testing in clinical practice for pancreatic tumors, it is important to highlight that a key concept in this framework is molecular redundancy [75,77]. This notion has been introduced and endorsed by all major professional organizations in the field, including the American Society of Clinical Oncology and the College of American Pathologists. Molecular laboratories should certify that test results that are unexpected, discordant, equivocal, or otherwise of low confidence should be confirmed or resolved using an alternative methodology [75,77]. Based on this assumption, we highlight that the possibility of performing an integrated approach for this important task appears as a crucial option in this context. In some cases, fusion genes can be easily detected by a type of analysis but also missed by another type [13,20].

Briefly, IHC is cost-effective and widely used in clinical practice. Compared with molecular techniques, IHC has few technical challenges and on appropriate validation with known fusion-positive neoplasms can be a powerful tool. However, the clinical utility of IHC is limited by available antibodies and is associated with false-positive and false-negative expression. As a result, orthogonal analysis, such as FISH testing, is often required to confirm or exclude a fusion event. FISH testing using dual-labeled, break-apart probes is broadly available and is considered the gold standard to detect fusion events without *a priori* knowledge of the fusion partner. Of note, break-apart probes cannot identify small intrachromosomal rearrangements or the exact fusion partner, and not all known DNA rearrangements produce an expressed fusion transcript. Moreover, FISH testing is limited to a single assay for each gene that is analyzed, and while multiplex testing has been reported, FISH testing requires expensive equipment and highly trained technical staff. Targeted DNA-based NGS allows the evaluation of multiple gene fusion breakpoints in combination with other pathological alterations, such as single nucleotide variants, small insertions/deletions, and copy number changes. The exact fusion genes must be known for targeted DNA-NGS, which limits the throughput of this technique. RNA-based NGS detects only expressed fusion genes and can discriminate between splicing isoforms, with quantification of fusion transcripts. It is not affected by intronic regions, but RNA extraction is more complicated than DNA, especially from formalin-fixed paraffin-embedded specimens as it can contain highly degraded nucleic acids that can invalidate fusion gene assessment. Nevertheless, RNA-NGS has detected novel fusion genes, but typically at least one partner gene must be known. Finally, RNA-seq allows complete assessment of known and unknown fusion gene analysis along with confirmation of RNA expression of the fusion event. The limitations of RNA-seq are similar to those encountered by RNA-NGS, but RNA-seq also requires increased technical expertise, such as complex bioinformatic analysis, to ensure the detection of low-expressing fusion events and to exclude false-positive fusion genes, such as stochastic gene rearrangements.

Fusion genes also have diagnostic potential in the context of pancreatic tumors. Fusions involving *PRKACA*/*B* genes were detected only in IOPN, characterizing all cases of this intraductal precursor. Such fusions can also be detected in the cyst fluid, thus representing a powerful diagnostic tool in preoperative settings [28]. Furthermore, because IOPN is associated with a favorable clinical course, even in cases of progression to infiltrative carcinoma [78], such fusions have prognostic significance. Finally, the detection of *FGFR2* fusions, albeit nonspecific, should consider not only PDAC, but also ITPN in the diagnostic workflow [79,80]. Indeed, they were typically detected in cancers derived from ITPN [18,54,79,80].

## Concluding remarks

Through systematic review and subsequent comparative analysis with the TCGA dataset, we have determined the low prevalence of fusion genes in pancreatic cancers/tumors, which stands at 1.2–1.4%. Although it is important to recognize that some questions remain unanswered in this complex scenario (see Outstanding questions), certain credible conclusions should also be acknowledged. First and foremost, this rare subgroup of patients almost uniformly stems from the group of *KRAS*-WT pancreatic cancers, which constitutes 8–12% of all pancreatic malignancies. Furthermore, fusion events are more common in ANs, ITPNs, and IOPNs. At present, fusion genes are the only detected genetic drivers in the majority of these tumors, highlighting their importance in tumor biology. Overall, fusion genes can play two distinct roles in the context of pancreatic cancer: (i) fusions involving *BRAF*, *FGFR2*, *RET*, *NTRK*, *NRG1*, and *ALK* should be investigated as potential actionable targets in precision oncology, especially for *KRAS*-WT PDAC, ANs, and invasive cancers derived from ITPN; and (ii) fusions involving *PRKACA*/*B* should be integrated into the diagnostic workflow for intraductal pancreatic neoplasms, representing the diagnostic hallmark of IOPNs.

Despite these conclusions, our study has some limitations. First, it depended on the different baseline characteristics of all patients in the analyzed studies. For example, some studies were specifically based on the detection of fusion genes and used integrated approaches with at least two different analyses to confirm the findings. Conversely, other studies applied only a single type of test for fusion detection, thus introducing a potential bias. Additionally, the types of studies were heterogeneous, ranging from retrospective investigations to clinical trials, with possible discrepancies in patient selection. Notably, to overcome the issue of patient selection, we specifically focused on studies with ‘real-world’ data to calculate the prevalence of fusion genes in pancreatic tumors/cancers. Furthermore, it may be possible that in our investigations intragenic rearrangements could also be considered along with classic fusion genes. However, this may be possible only if the ultimate result was an oncogenic activation identical to gene fusion. Finally, not all studies reported complete data regarding age, sex, tumor histology, tumor location in the pancreas, and specific molecular background; however, the overall sample size appeared to be adequate to support reliable conclusions.

In this complex scenario, new clinical trials testing existing and novel therapeutic agents for fusion genes in pancreatic cancers are urgently needed, also considering the different histologies and molecular background. New studies should also investigate the optimal timing for the administration of fusion-based targeted therapies. Furthermore, dissection of the tumor microenvironment with spatial profiling technologies and understanding its impact in precision oncology represent critical challenges in pancreatic cancer harboring fusion genes [81]. That said, there is an urgent need for quality-ensured molecular diagnostics in the management of pancreatic tumors.

## Supplementary Material

Supplementary Material

Supplemental information

Supplemental information associated with this article can be found online at https://doi.org/10.1016/j.trecan.2024.01.009

## Figures and Tables

**Table 1.** Summary of the most important clinicopathological and molecular data for pancreatic tumors with fusion genes^a^

Study	Cases (detected gene fusions)	Gender	Age (years)	Histology	Location	Assay	*KRAS* status	Other relevant molecular alterations
61 studies	Total number of tumors investigated for fusions: 17 044 Total number of fusions in pancreatic tumors: 339 Total number of cancers investigated for fusions: 16 845 Total number of fusions in pancreatic cancers: 263 Real prevalence of fusions in tumors: 242/16 945 (1.4%) Real prevalence of fusions in cancers: 208/16 802 (1.2%)	143 M 82 F	Mean 57.30, range 7–86, *σ* 16.81	Total fusions in PDAC: 206 Real prevalence of fusions in PDAC: 144/11 702 (1.2%) Total fusions in AN: 53 Real prevalence of fusions in AN: 33/167 (19.8%) Total fusions in IOPN: 39 Real prevalence of fusions in IOPN: 39/39 (100%) Total fusions in ITPN: 29 Real prevalence of fusions in ITPN: 29/57 (50.9%) Fusions in other cases: 7 NEN, 1 MiNEN, 4 IPMN	49 H (51.6%), 16 B (16.8%), 9 T (9.5%), 3 H+B (3.2%), 10 B+T (10.5%), 8 H+B+T (8.4); 60 involving H (63.2%), 35 not involving H (36.8%)	22 DNA-NGS, 6 RNA-NGS, 18 DNA/RNA-NGS, 5 DNA/RNA-NGS/FISH, 1 NGS NOS/FISH/PCR, 1 NGS NOS/PCR, 1 FISH, 4 DNA-NGS/FISH, 3 RNA-NGS/FISH	*KRAS* status when determined: WT 283/289 (98%), mut 6/289 (2%); *KRAS* status in cancers: WT 180/182 (98.9%), mut 2/182 (1.1%)	Total cases with such information: 238; no co-occurring alterations: 138/238 (58.0%); co-occurring alterations: 100/238 (42.0%); for only cancers: no co-occurring alterations: 89/162 (54.9%); co-occurring alterations: 73/162 (45.1%) PDAC: 68/129 no other alterations; recurrent: 24 *TP53* mut, 17 *CDKN2A* and/or *CDKN2B* HD, 12 *SMAD4* mut/HD, 7 *GNAS* mut, 6 *MCL1* amp, 5 *MYC* amp, 2 *BRCA2* mut, 2 *PI3KCA* mut AN: 21/33 no other alterations; recurrent: 9 *CDKN2A* and/or *CDKN2B* HD, 5 *SMAD4* mut/HD, 2 *TP53* mut IOPN: 37/39 no other alterations; 1 *BRAF* mut, 1 *ERBB2* mut ITPN: 8/29 no other alterations; recurrent: 9 *SMAD4* mut/HD, 8 *TP53* mut/HD, 7 *CDKN2A* and/or *CDKN2B* HD, 3 *MYC* amp

a Abbreviations: amp, amplification; B, body of the pancreas; H, head of the pancreas; MiNEN, mixed neuroendocrine-non-neuroendocrine neoplasm; mut, mutated; NEN, neuroendocrine neoplasm; T, tail of the pancreas.

**Table 2.** Summary of potentially druggable fusion genes identified in pancreatic tumors^a,f^

Main fusion gene	*ALK*	*BRAF*	*FGFR2*	*MET*	*NRG1*	*NTRK1/2/3*	*RAF1*	*RET*	*ROS1*
Partner	*ALK* fusion (6)^b^ *EML4* (10) *STRN* (6) *PPF1BP1* (2) *DNCT1* (1) *KANK4* (1) *ERC1* (1) *ALK* rearrangement (1)^b^	*SND1* (30) *BRAF* fusion (16)^b^ *BRAF* rearrangement (11)^b^ *MYRIP* (3) *TRIM24* (3) *GIPC2* (3) *MKRN1* (2) *MBNL2* (2) *TNS3* (2) *AGK* (2) *HERPUD1* (2) *AGAP3* (1) *CUX1* (1) *RPS25* (1) *SRPRA* (1) *NPHP3* (1) *C16orf72* (1) *RELN* (1) *LUC7L2* (1) *EPS15L1* (1) *GLI2* (1) *JHDM1D* (1) *STRN3* (1) *SVOPL* (1) *TMEM9* (1) *FRY* (1) *ZSCAN30* (1) *GATM* (1) *TRIM44* (1)	*FGFR2* fusion (12)^b^ *TACC2* (3) *POC1B* (2) *VCL* (2) *INA* (2) *USP33* (1) *CCSER2* (1) *CGNL1* (1) *KCTD1* (1) *BICC1* (2) *CIT* (1) *FAM76A* (1) *PPHLN1* (1) *NOL4* (1) *KIAA1524* (1) *CCDC170* (1) *CEP112* (1) *TRIM8* (1) *KIAAA1217* (1) *MAST4* (1) *COBLL1* (1) *HSP17B4* (1) *SYCP1* (1) *FGFR2* rearrangement (2)^b^ *CEP55* (1) *SASS6* (1) *CREB5* (1) *SHROOM3* (1) *DISP1* (1) *TXLNA* (1) *GCC2* (1)	*MET* fusion (2)^b^ *TNS3* (2) *RDX* (1)	*ATP1B1* (12) *NRG1* fusion (3)^b^ *APP* (3) *SARAF/CDH6* (1) *SLC3A2* (1) *CDH1* (1) *VTCN1* (1)	*NTRK* fusion (6)^b^ *TPR* (3) *EML4* (2) *KANK1* (1) *THAP1* (1) *SEL1L* (1) *CTRC* (1) *NOS1AP* (1) *ERC1* (1)	*PDZRN3* (5) *RAF1* fusion (3)^b^ *IQSEC1* (2) *GATM* (1) *KANK4* (2) *GOLGA4* (2) *HERPUD1* (1) *TRIM33* (1) *ALCAM* (1) *AGGF1* (1) *SLC43A1* (1) *FGD5* (1) *JAK1* (1) *HACL1* (1)	*RET* fusions (14)^b^ *CCDC6* (9) *TRIM33* (2) *TRIM24* (2) *ERC1* (2) *RET* rearrangement (2)^b^ *JMJD1C* (1) *NCOA4* (2) *SATB1* (1) *C14orf93* (1)	*ROS1* fusion (2)^b^ *SLC4A4* (2) SLC34A2 (1)
Subtype									
PDAC	25	48	32	5	19	8	13	23	3
PACC^c^	1	37	–	–	–	1	8	4	–
PC *NOS*	–	1	2	–	1	8	1	5	1
ITPN/PDAC	1	2	7	–	2	–	–	2	–
IPMN/PDAC	–	4	–	–	–	–	–	–	–
ITPN/PC	1	–	–	–	–	–	–	–	–
*NOS*	–	–	6	–	–	–	–	1	–
ITPN	–	–	1	–	–	–	1	–	1
PB	–	1	–	–	–	–	–	–	–
Other									
Gender									
Male	16	39	17	1	8	6	12	10	1
Female	4	24	10	1	5	2	6	5	2
Age (years) (median)	39.5	62.0	62.0	67.5	50.0	51.0	60.0	62.5	54.0
Response									
CR^d^	–	–	2	1	–	2	–	1	–
PR^e^	6	1	2	–	6	3	3	10	1
R, *NOS*	–	–	–	–	–	3	–	–	–
SD	4	2	–	–	3	1	1	–	1
PD	2	1	–	–	–	–	1	–	–
Int.	–	1	1	–	–	–	–	–	–

a Abbreviations: CR, complete response; Int., intolerance; IPMN, intraductal papillary mucinous neoplasm; NET, neuroendocrine tumor; PACC, pancreatic acinar cell carcinoma; PB, pancreatoblastoma; PC, pancreatic cancer; PD, progressive disease; PR, partial response; R, response; SD, stable disease.

b The fusion gene is not otherwise specified (partner gene is not specified).

c PACC category also includes mixed PACC/PDAC/NET.

d Patients with exceptional response are included here.

e Patients with significant response are included here.

f The number between brackets beside each gene name represents the number of cases harboring the specific gene fusion.

**Table 3.** Summary of patients’ response to targeted agents based on specific fusion genes (cancer stage is also reported)^a,e^

Main fusion gene	CR^b^ (no of cases^c^)^Stage at diagnosis, targeted agent^	PR^d^ (no of cases)^Stage at diagnosis, targeted agent^	R, *NOS* (no of cases)^Stage at diagnosis, targeted agent^	SD (no of cases)^Stage at diagnosis, targeted agent^	PD (no of cases)^Stage at diagnosis, targeted agent^	Int. (no of cases)^Stage at diagnosis, targeted agent^
*ALK*	–	6 patients^II B,1; NO S,1; IV, 2+3; II B,2; III, 1+2; IV,2+3e^	–	4 patients^I V,1; IV, 1/2; I V,2; IV,4/2^	2 patients^IV,1+2; IV,2^	–
*BRAF*	–	1 patient^IV,5^	–	2 patients^IV,5; IV,5^	1 patient^IV,6^	1 patient^IV,5+7^
*FGFR2*	2 patients^IV,8; IV,8^	2 patients^IV,8; NOS,9^	–	–	–	1 patient^IV,10^
*MET*	1 patient^NOS,1^	–	–	–	–	–
*NRG1*	–	6 patients^IV,12 +14; IV ,13; IV ,13; IV ,13; IIB ,16; IV,17^	–	3 patients^IV,12+15; NOS,13; IV,16^	–	–
*NTRK1/2/3*	2 patients^IIB,18; IV,18^	3 patients^NOS,11; IV,18+19; NOS,18^	3 patients^LA-IV,11; LA-IV,11; LA- IV,11^	1 patient^IV,11^	–	–
*RAF1*	–	3 patients^IV,5; IV,5; IV,20+21^		1 patient^NOS,5+7^	1 patient^IV,5^	–
*RET*	1 patient^IV,22^	10 patients^NOS,22; NOS,22; IV,22; IV,22; NOS,23; NOS,23; NOS,23; NOS,23; NOS,23; NOS,23^	–	–	–	–
*ROS1*	–	1 patient^IV,1+3^	–	1 patient^IV,11^	–	–
Total	6 patients	32 patients	3 patients	12 patients	4 patients	2 patients

a Abbreviations: CR, complete response; Int., intolerance; LA, locally advanced; PD, progressive disease; PR, partial response; R, response; SD, stable disease; –, not applicable.

b Patients with exceptional response are included here.

c After the number of patients, we have indicated tumor stage (if present in the original manuscript) and the targeted agent (superscript).

d Patients with significant response are included here.

e Targeted agent information: ALK inhibitors: crizotinib^1^, alectinib^2^, lorlatinib^3^, ceritinib^4^; MEK inhibitor: trametinib^5^; RAF inhibitor: pan-RAF inhibitor *NOS*^6^; BRAF inhibitor: dabrafenib^7^; FGFR1-4 inhibitor: erdafitinib^8^; FGFR1-3 inhibitor: pemigatinib^9^; tyrosine kinase inhibitor: nintedanib^10^, entrectinib^11^; EGFR TKI: erlotinib^12^; HER inhibitor: afatinib^13^; anti-HER2 antibody: pertuzumab^14^, trastuzumab^15^; anti-HER2/HER3 bispecific antibody: zenocutuzumab^16^; anti-HER3 antibody: seribantumab^17^; NTRK inhibitors: larotrectinib^18^, selitrectinib^19^; multikinase inhibitors: sorafenib^20^, regorafenib^21^; RET inhibitors: pralsetinib^22^, selpercatinib^23^.

**Table 4.** Advantages and limitations of the different testing modalities for the detection of fusion genes

Parameter	IHC	FISH	Targeted DNA-based NGS	Targeted RNA-based NGS	Whole transcriptome sequencing (RNA-seq)
Turn-around time	Low	Low	Medium	Medium	High
Input material	Low	Low	Low–medium	Medium	High
Costs	Low	Medium	Medium–high	Medium–high	High
Specific annotation of variants	No	No	Yes	Yes	Yes
Sensitivity/specificity	Medium/medium	Medium/high	High/high	Very high/very high	Very high/very high
Advantages	Fast, cost-effective, and widely available	Highly specific and allows direct visualization of fusion events	High accuracy that facilitates detection of known fusion genes	High accuracy that facilitates detection of known fusion genes	High sensitivity and the ability to discover novel fusion events that can also quantify gene expression levels
Challenges	Nonspecific staining can lead to false-positive findings	Requires specialized equipment and technical staff; may not detect novel fusion genes and can be cost prohibitive	Limited throughput and inability to detect novel fusion genes	Limited to known fusion partners and low sensitivity for novel fusion genes	Requirement for high-quality RNA, potential false positives, and difficulties in detection of low-expression fusion genes

## Highlights

Using a systematic review-based approach, we explored the potential roles of fusion genes in pancreatic tumors.

The prevalence of fusion genes in pancreatic tumors is low (1.4%), but they are typically enriched in *KRAS* wild-type (WT) tumors (98% of cases).

Fusion genes may represent actionable targets in the context of precision oncology and diagnostic biomarkers for pancreatic intraductal neoplasms.

Fusions involving *BRAF*, *FGFR2*, *RET*, *NTRK*, *NRG1*, and *ALK* are actionable targets in *KRAS*-WT pancreatic ductal adenocarcinomas and acinar neoplasms.

Fusions involving *PRKACA*/*B* are a diagnostic hallmark of intraductal oncocytic papillary neoplasms.

## Outstanding questions

What is the most effective methodology for the detection of gene fusions in cancer in general and in pancreatic cancer in particular?

Does the tissue microenvironment (stroma and immune cells) influence the response to agents targeting specific cancer cell fusion genes?

What is the optimal timing for precision oncology based on specific fusion genes in the clinical history of patients with pancreatic cancer?

In cases where actionable targets (e.g., MSI) and fusion genes coexist, what is the optimal strategy to determine the most suitable treatment for patients?

Should specific gene fusions be included in the standard assays for the molecular diagnosis of pancreatic neoplasms?

## Glossary

Acinar neoplasms (ANs): rare pancreatic neoplasms arising from the exocrine pancreas and accounting for approximately 1–2% of all pancreatic tumors. They include acinar cell carcinoma and pancreatoblastoma.
Fusion gene: a hybrid gene generated by two independent genes usually located in close proximity in a chromosome, resulting into a chimeric protein containing features of both genes. In some occasions, such a fusion gene functions as an oncogene, representing an actionable target for precision oncology.
Pancreatic ductal adenocarcinoma (PDAC): the most common subtype of pancreatic cancer, responsible for more than 90% of all deaths from pancreatic malignancies. It has a very aggressive biological behavior and very rarely harbors molecularly actionable targets.
Precision oncology: an innovative approach to cancer treatment that ensures your therapy is designed and tailored to your specific form of cancer. It is based on the molecular profiling of tumors to identify actionable targets based on genomic alterations.
The Cancer Genome Atlas (TCGA): a publicly accessible cancer database with a comprehensive dataset of molecular data (genomics, epigenomics, transcriptomics, proteomics) derived from the characterization of thousands of samples from 33 cancer types.
Tumor-agnostic treatment: a type of targeted therapy that can be administrated in patients with cancer harboring specific molecular alterations, regardless of tumor type/histology.
