# Supplementary material for: Fusion genes in pancreatic tumors
Source: Trends Cancer. Author manuscript; Available in PMC 2026 Aug 30. (PMC13526370; doi:10.1016/j.trecan.2024.01.009)

SUPPLEMENTARY MATERIAL

Supplementary Methods

Systematic review of the literature: search strategy and criteria for selecting the studies

Two investigators (A.G. and C.L.) independently conducted a literature search using PubMed, SCOPUS, and Embase, without language restrictions, from database inception until July 2023 for all published studies providing original data on fusion genes in pancreatic tumors.

The following search-strategy was used: ("fusion" OR "fusions" OR "fusion-gene" OR "fusion-genes") AND ("pancreatic cancer" OR "pancreatic cancers" OR "pancreatic tumor" OR "pancreatic tumors" OR "pancreatic tumour" OR "pancreatic tumours" OR "pancreatic neoplasm" OR "pancreatic neoplasms" OR "pancreatic carcinoma" OR "pancreatic carcinomas" OR "pancreatic adenocarcinoma" OR "pancreatic adenocarcinomas"). Following the searches as outlined above, after removal of duplicates, two independent reviewers (A.G. and C.L.) screened titles and abstracts of all potentially eligible articles. The two authors applied the eligibility criteria, considered the full texts and a final list of included articles was reached through consensus.

The reference lists of pertinent reviews were manually searched to identify additional potentially relevant articles. The exclusion criteria were as follows: 1) no original cases/original information, 2) no data on pancreatic tumors, 3) extra-pancreatic and/or non-epithelial origin, 4) no full-text, and 5) *in vitr*o or animal studies. In cases of double cohorts/patients, we selected the study with the most complete information and/or the largest number of cases and/or the largest number of cases with clinicopathological data.

Data extraction, synthesis, and analysis

Two investigators (A.G. and C.L.) extracted key data from the included articles and a third independent investigator (A.S.) checked the data.

For each article, we extracted the following information: number of patients with pancreatic tumors analyzed for any gene fusion, number of tumors with gene fusion, patient age, sex, tumor histology, tumor location in the pancreas, type of assay for detecting gene fusion, administration of targeted therapy based on gene fusion and related response/survival (also interpreting data from figures, if needed), *KRAS* status, and the presence of co-occurring molecular alterations

(**Supplementary Table 1**). The most relevant data are summarized in **Table 1**. We also described all detected actionable (**Table 2**) and non-druggable (**Supplementary Table 2**) gene fusions in pancreatic tumors. We considered as cancers all infiltrative tumors of epithelial origin (e.g.: pancreatic cancer not otherwise specified, pancreatic ductal adenocarcinoma (PDAC), acinar cell carcinoma, pancreatoblastoma), as per WHO guidelines.

To assess differences in the molecular landscape among cases harboring different fusion genes, and to differentiate between pancreatic cancer in unselected patients versus those with fusion genes, we used Fisher's exact test. This was utilized to juxtapose the findings with data from The Cancer Genome Atlas Research Network (TCGA, https://www.cancer.gov/ccg/research/genome-sequencing/tcga, last access 09/15/2023), which we used as a reference cohort.

Identification of manuscripts

The literature search identified 776 unduplicated articles; 588 were excluded after reviewing their titles and abstracts, resulting in 188 articles eligible for full-text review. After applying our inclusion criteria, 61 articles were finally included in the systematic review (**Supplementary Figure 1**).

Supplementary Table 1. PRISMA research checklist

TITLE	#	Checklist item	Reported on page #
Title	1	Identify the report as a systematic review, meta-analysis, or both.	3
**ABSTRACT**			
(Structured) summary	2	Provide a structured summary including, as applicable: background; objectives; data sources; study eligibility criteria, participants, and interventions; study appraisal and synthesis methods; results; limitations; conclusions and implications of key findings; systematic review registration number.	2
**INTRODUCTION**			
Rationale	3	Describe the rationale for the review in the context of what is already known.	3
Objectives	4	Provide an explicit statement of questions being addressed with reference to participants, interventions, comparisons, outcomes, and study design (PICOS).	3
**METHODS**			
Protocol and registration	5	Indicate if a review protocol exists, if and where it can be accessed (e.g., Web address), and, if available, provide registration information including registration number.	-
Eligibility criteria	6	Specify study characteristics (e.g., PICOS, length of follow-up) and report characteristics (e.g., years considered, language, publication status) used as criteria for eligibility, giving rationale.	4
Information sources	7	Describe all information sources (e.g., databases with dates of coverage, contact with study authors to identify additional studies) in the search and date last searched.	4
Search	8	Present full electronic search strategy for at least one database, including any limits used, such that it could be repeated.	4
Study selection	9	State the process for selecting studies (i.e., screening, eligibility, included in systematic review, and, if applicable, included in the meta-analysis).	4
Data collection process	10	Describe method of data extraction from reports (e.g., piloted forms, independently, in duplicate) and any processes for obtaining and confirming data from investigators.	4
Data items	11	List and define all variables for which data were sought (e.g., PICOS, funding sources) and any assumptions and simplifications made.	4-5
Risk of bias in individual studies	12	Describe methods used for assessing risk of bias of individual studies (including specification of whether this was done at the study or outcome level), and how this information is to be used in any data synthesis.	4-5
Summary measures	13	State the principal summary measures (e.g., risk ratio, difference in means).	4-5
Synthesis of results	14	Describe the methods of handling data and combining results of studies, if done, including measures of consistency (e.g., I^2) for each meta-analysis.	-

Risk of bias across studies	15	Specify any assessment of risk of bias that may affect the cumulative evidence (e.g., publication bias, selective reporting within studies).	-
Additional analyses	16	Describe methods of additional analyses (e.g., sensitivity or subgroup analyses, meta-regression), if done, indicating which were pre-specified.	5
**RESULTS**			
Study selection	17	Give numbers of studies screened, assessed for eligibility, and included in the review, with reasons for exclusions at each stage, ideally with a flow diagram.	5
Study characteristics	18	For each study, present characteristics for which data were extracted (e.g., study size, PICOS, follow-up period) and provide the citations.	5-7
Risk of bias within studies	19	Present data on risk of bias of each study and, if available, any outcome level assessment (see item 12).	-
Results of individual studies	20	For all outcomes considered (benefits or harms), present, for each study: (a) simple summary data for each intervention group (b) effect estimates and confidence intervals, ideally with a forest plot.	-
Synthesis of results	21	Present results of each meta-analysis done, including confidence intervals and measures of consistency.	-
Risk of bias across studies	22	Present results of any assessment of risk of bias across studies (see Item 15).	-
Additional analysis	23	Give results of additional analyses, if done (e.g., sensitivity or subgroup analyses, meta-regression [see Item 16]).	5-7
**DISCUSSION**			
Summary of evidence	24	Summarize the main findings including the strength of evidence for each main outcome; consider their relevance to key groups (e.g., healthcare providers, users, and policy makers).	7-8
Limitations	25	Discuss limitations at study and outcome level (e.g., risk of bias), and at review-level (e.g., incomplete retrieval of identified research, reporting bias).	10
Conclusions	26	Provide a general interpretation of the results in the context of other evidence, and implications for future research.	8-10
**FUNDING**			
Funding	27	Describe sources of funding for the systematic review and other support (e.g., supply of data); role of funders for the systematic review.	18

Adapted from: Moher D, Liberati A, Tetzlaff J, Altman DG, The PRISMA Group (2009). Preferred Reporting Items for Systematic Reviews and Meta-Analyses: The PRISMA Statement. PLoS Med 6(6): e1000097. doi:10.1371/journal.pmed1000097. For more information, visit: www.prisma-statement.org.

Supplementary Table 2. Summarizing table (study by study) of clinicopathological and molecular information of pancreatic tumors with fusion genes.

Study	Cases (Detected Gene Fusions)	Gender	Age	Histology	Location	Assay	Gene Fusion	Targeted agent	*KRAS* status	Other relevant molecular alterations	Response[a]/ Survival[b]
Chmielecki et al.[13]	44 (10)	M (3) NA (3)	59, 70, 79, NA (3)	PACC (2) Mixed PACC+PDAC (4)	NA	NGS DNA[1]/RNA[2]	2 x *SND1 (1-10)::BRAF (9-18)*	NA	WT	None	NA
					NA	NGS DNA[1]/RNA[2]	*SND1 (1-14)::BRAF (9-18)*	NA	WT	None	NA
					NA	NGS DNA[1]/RNA[2]	*SND1 (1-9)::BRAF (9-18)*	NA	WT	*TP53* mutation *CDKN2A/B HD*	NA
					NA	NGS DNA[1]/RNA[2]	*SND1 (1-10)::BRAF (11-18)*	NA	WT	None	NA
					NA	NGS DNA[1]/RNA[2]	*SND1 (1-14)::BRAF (11-18)*	NA	WT	*CDKN2A/B HD*	NA
		M	69	PACC	NA	FISH, NGS DNA[1]/RNA[2]	*HERPUDI (1-4)::BRAF (7-18)*	NA	WT	None	NA
		NA	NA	Mixed PACC+NEN	NA	FISH, NGS DNA[1]/RNA[2]	*ZSCAN30 (1-3)::BRAF (10-18)*	NA	WT	None	NA

		M	76	PACC	NA	FISH, NGS DNA[1]	*GATM (1-2)::BRAF (11-18)*	NA	WT	*SMAD4* mutation *CDKN2A/B* HD	NA
		NA	NA	Mixed PACC+PDAC	NA	NGS DNA[1]/RNA[2]	*HACL1 (1-16)::RAF1 (8-17)*	NA	WT	None	NA
Ross et al.[14]	1062 (3)	M	63	PDAC	NA	FoundationOne[3]	*MYRIP (16)::BRAF (9)*	NA	WT	NA	NA
		F	75	PACC	NA	FoundationOne[3]	*SND1 (10)::BRAF (9)*	NA	WT	NA	NA
		M	67	PACC	NA	FoundationOne[3]	*SND1 (10)::BRAF (9)*	NA	WT	NA	NA
Shimada et al.[15]	100 (1)	F	72	PDAC	Head	WTS, RT-PCR, Sanger sequencing	*DNCT1 (12)::ALK (20)*	NA	WT	*TP53* mutation *CDKN2A* mutation *GNAS* mutation	NA
Scarpa et al.[16]	98 (3)	M	60	PanNET G3	NA	WGS	*EWSR1::BEND2*	NA	WT	NA	NA
		F	30	PanNET G2	NA	WGS	*EWSR1::BEND2*	NA	WT	NA	NA
		M	38	PanNET G3 (Poorly differentiated)	NA	WGS, RNA sequencing	*EWSR1 (7)::FLI1 (6)*	NA	WT	NA	NA

Singhi et al.[17]	3.170 (5)	M	35	Tubular-type PDAC	Head	NGS DNA[4], FISH, IHC	*EML4 (13)*:: *ALK (20)*	Ceritinib/ Alectinib	WT	*BAP1* mutation *NFE2L2* mutation *MCL1* amp	SD for 7 months (Alive)
		F	32	Tubular-type PDAC with mucinous features	Head	NGS DNA[4]	*EML4 (6)::* *ALK (20)*	Crizotinib	WT	*CDKN2A*/*B* HD *MYC* amp *TP53* mutation	SD for 17 months/Dead of septic shock
		M	34	Tubular-type PDAC	Head	NGS DNA[4], FISH, IHC	*STRN (3)::* *ALK (20)*	EBRT+ Gemcitabine +Crizotinib /Crizotinib	WT	None	PR for 2 months (Alive)
		M	46	Tubular-type PDAC	Head	NGS DNA[4]	*EML4 (6)::* *ALK (20)*	Crizotinib/ Alectinib	WT	*CDKN2A*/*B* HD *TP53* mutation *SMAD4* mutation *FGFR1* mutation	PD after 2 months (Alive)
		M	43	Colloid Carcinoma[5]	NA	NGS DNA[4]	*EML4 (6)::* *ALK (20)*	NA	WT	None	NA
Basturk et al.[18]	18 (5)	NA	NA	ITPN	NA	MSK-IMPACT[6], Ion Ampliseq Comprehensive Cancer Panel[7], WES, WGS	*FGFR2::CEP55*	NA	WT	None	NA
		NA	NA	ITPN	NA	MSK-IMPACT[6], Ion Ampliseq Comprehensive Cancer Panel[7], WES	*FGFR2::SASS6*	NA	WT	None	NA

		NA	NA	ITPN	NA	MSK-IMPACT[6], Ion Ampliseq Comprehensive Cancer Panel[7], WES	*FGFR2::DISP1* *FGFR2::TXLNA*	NA	WT	None	NA
		NA	NA	ITPN	NA	MSK-IMPACT[6], Ion Ampliseq Comprehensive Cancer Panel[7], WES	*VCL-FGFR2*	NA	WT	None	NA
		NA	NA	ITPN/PC *NOS*	NA	MSK-IMPACT[6], Ion Ampliseq Comprehensive Cancer Panel[7], WES, Archer FusionPlexTM Solid Panel[8]	*STRN::ALK*	NA	WT	None	NA
Tuli et al.[19]	1 (1)	M	34	PDAC	Head	FoundationOne[3], IHC	*STRN (1-3):: ALK (20-29)*	IMRT+ Gemcitabine +Crizotinib/ Crizotinib	WT	None	PR for 7 months (Alive)
Wang et al.[20]	18 (5)[9]	M	79	PACC	NA	FISH, NGS DNA[1]/RNA[2]	*BRAF* fusion *NOS*	NA	NA	NA	NA
		F	45	PACC	NA	FISH, NGS DNA[1]/RNA[2]	*SND1 (10)::BRAF (9)*	NA	NA	NA	NA
		F	70	PACC	NA	FISH, NGS DNA[1]/RNA[2]	*HERPUDI (7)::BRAF (7)*	NA	NA	NA	NA

		M	69	Mixed PACC+NEN	NA	FISH, NGS DNA[1]/RNA[2]	*GIPC2 (3)::BRAF (10)*	NA	NA	NA	NA
		M	46	Mixed PACC+NEN +PDAC	NA	FISH, NGS DNA[1]/RNA[2]	*SND1 (9)::BRAF (9)*	NA	NA	NA	NA
Heining et al.[21]	17 (4)	F	29	PDAC	NA	WGS, RNA sequencing[10]	*ATP1B1::NRG1*	Afatinib	WT	*SMAD4* mutation	Remission of liver met at $1^{3/4}$ months. PD after $4^{3/4}$ months
		F	39	PDAC	NA	WES, RNA sequencing[10]	*SARAF::NRG1 ::CDH6*	Erlotinib+ Pertuzumab	WT	None	Partial remission of liver met at 2 months. PD after 5 months
		M	24	PDAC	NA	WGS	*APP::NRG1*	Erlotinib+ Trastuzumab +CHT[c]	WT	None	SD for 3 months
		M	38	PDAC	NA	WGS, RNA sequencing[10]	*NCOA4::RET*	NA	WT	*CDKN2A/B* HD	NA
Pishvaian et al.[22]	3 (3)	M	51	PC *NOS*	NA	NGS[11], FISH, IHC	*TPR::NTRK1*	Entrectinib	WT	None	SD for 13 months (Alive)
		M	47	PDAC	NA	FoundationOne[3]	*TPR::NTRK1*	Entrectinib	WT	*CDKN2A/B* HD	PR for 8 months. Then PD (Alive)

		M	54	PC *NOS*	NA	MSK-IMPACT[6]	*SLC4A4*::*ROS1*	Entrectinib	WT	*CDKN2A* mutation *BRCA2* mutation	SD for 7 months. Then PD (Alive)
Singhi et al.[23]	3594 (51)	M	78	PDAC	NA	NGS DNA[12]	*CUX1(18)::BRAF (7)*	NA	WT	*TP53* mutation *GNAS* mutation	NA
		M	77	PDAC	NA	NGS DNA[12]	*RPS25(3)::BRAF (9)*	NA	WT	*TP53* mutation	NA
		F	51	PDAC	NA	NGS DNA[12]	*MBNL2 (8)::BRAF (9)*	NA	WT	*BRCA2* mutation	NA
		F	83	PDAC	NA	NGS DNA[12]	*SRPRA (4)::BRAF (10)*	NA	WT	*TP53* mutation *MYC* amp	NA
		F	69	PDAC	NA	NGS DNA[12]	*NPHP3 (4)::BRAF (10)*	NA	WT	*MCL1* amp *NTRK1* amp	NA
		F	56	PDAC	NA	NGS DNA[12]	*C16orf72 (3)::BRAF (10)*	NA	WT	None	NA
		NA	NA	PDAC	NA	NGS DNA[12]	*FRY (55)::BRAF (10)*	NA	WT	None	NA

		F	44	PDAC	NA	NGS DNA[12]	*MKRN1 (4)::BRAF (11)*	NA	WT	*CDK4* amp	NA
		M	66	PDAC	NA	NGS DNA[12]	*SND1 (9)::BRAF (9)*	NA	WT	None	NA
		M	75	PDAC	NA	NGS DNA[12]	*SND1 (10)::BRAF (9)*	NA	WT	None	NA
		M	50	PDAC	NA	NGS DNA[12]	*SND1 (10)::BRAF (11)*	NA	WT	None	NA
		F	56	PDAC	NA	NGS DNA[12]	*SND1 (11)::BRAF (11)*	NA	WT	*MCL1* amp	NA
		NA	NA	PDAC	NA	NGS DNA[12]	*SND1::BRAF*	NA	WT	*ATM* mutation *MCL1* amp	NA
		M	55	PDAC	NA	NGS DNA[12]	*RELN::BRAF*	NA	WT	*CDKN2A* mutation *GNAS* mutation	NA
		M	61	PDAC	NA	NGS DNA[12]	*MYRIP::BRAF*	NA	WT	*RB1* mutation *GNAS* mutation	NA

		NA	NA	PDAC	NA	NGS DNA[12]	*GIPC2::BRAF*	NA	WT	None	NA
		M	49	PDAC	NA	NGS DNA[12]	*FGFR2 (17)::KIAA1524 (15)*	NA	WT	None	NA
		M	74	PDAC	NA	NGS DNA[12]	*FGFR2 (17)::CCDC170 (10)*	NA	WT	None	NA
		M	64	PDAC	NA	NGS DNA[12]	*FGFR2 (17)::CEP112 (17)*	NA	WT	*TP53* mutation	NA
		NA	NA	PDAC	NA	NGS DNA[12]	*FGFR2 (17)::POC1B (11)*	NA	WT	*TP53* mutation	NA
		M	47	PDAC	NA	NGS DNA[12]	*FGFR2 ::POC1B*	NA	WT	None	NA
		M	26	PDAC	NA	NGS DNA[12]	*FGFR2 (17)::TRIM8 (2)*	NA	WT	None	NA
		M	71	PDAC	NA	NGS DNA[12]	*FGFR2 (17)::KIAA1217 (2)*	NA	WT	None	NA

		NA	NA	PDAC	NA	NGS DNA[12]	*FGFR2 (17)::SHROOM 3 (6)*	NA	WT	*FGFR2* mutation	NA
		F	57	PDAC	NA	NGS DNA[12]	*FGFR2 (18)::BICC1 (3)*	NA	WT	None	NA
		M	57	PDAC	NA	NGS DNA[12]	*FGFR2 (18)::MAST4 (3)*	NA	WT	None	NA
		M	57	PDAC	NA	NGS DNA[12]	*FGFR2 (18)::VCL (1)*	NA	WT	*PTEN* mutation	NA
		M	30	PDAC	NA	NGS DNA[12]	*FGFR2 (18)::COBLL1 (12)*	NA	WT	None	NA
		M	61	PDAC	NA	NGS DNA[12]	*ALCAM (14)::RAF1 (6)*	NA	WT	None	NA
		M	67	PDAC	NA	NGS DNA[12]	*AGGF1 (1)::RAF1 (8)*	NA	WT	*TP53* mutation	NA
		M	64	PDAC	NA	NGS DNA[12]	*PDZRN3 (5)::RAF1 (8)*	NA	WT	*TP53* mutation	NA

		M	59	PDAC	NA	NGS DNA[12]	*PDZRN3 (7)::RAF1 (8)*	NA	WT	None	NA
		M	72	PDAC	NA	NGS DNA[12]	*GOLGA4 (11)::RAF1 (8)*	NA	WT	None	NA
		F	55	PDAC	NA	NGS DNA[12]	*SLC43A1::RAF 1*	NA	WT	None	NA
		F	60	PDAC	NA	NGS DNA[12]	*FGD5::RAF1*	NA	WT	*PI3KCA* mutation	NA
		M	34	PDAC	NA	NGS DNA[12]	*STRN (3)::ALK (20)*	NA	WT	None	NA
		M	36	PDAC	NA	NGS DNA[12]	*EML4 (6)::ALK (20)*	NA	WT	*MCL1* amp	NA
		M	45	PDAC	NA	NGS DNA[12]	*EML4 (13)::ALK (20)*	NA	WT	*TP53* mutation *SMAD4* mutation	NA
		M	38	PDAC	NA	NGS DNA[12]	*EML4::ALK*	NA	WT	*MCL1* amp	NA

		NA	NA	PDAC	NA	NGS DNA[12]	*ERC1 (11)::ALK (20)*	NA	WT	None	NA
		M	53	PDAC	NA	NGS DNA[12]	*CCDC6 (1)::RET (10)*	NA	WT	*TP53* mutation<br>*GNAS* mutation	NA
		F	75	PDAC	NA	NGS DNA[12]	*CCDC6 (1)::RET (12)*	NA	WT	None	NA
		NA	NA	PDAC	NA	NGS DNA[12]	*CCDC6::RET*	NA	WT	None	NA
		F	61	PDAC	NA	NGS DNA[12]	*SATB1 (7)::RET (12)*	NA	WT	None	NA
		NA	NA	PDAC	NA	NGS DNA[12]	*ERC1 (16)::NTRK1 (11)*	NA	WT	*TP53* mutation<br>*MYC* amp	NA
		M	45	PDAC	NA	NGS DNA[12]	*TPR (21)::NTRK1 (12)*	NA	WT	None	NA
		F	58	PDAC	NA	NGS DNA[12]	*NUMA1 (13)::ERBB4 (18)*	NA	WT	None	NA

		M	63	PDAC	NA	NGS DNA[12]	*FGFR3 (17)::TACC3 (11)*	NA	WT	*TP53* mutation *CTNNB1* mutation	NA
		F	58	PDAC	NA	NGS DNA[12]	*EGFR (24)::SEPT14 (10)*	NA	WT	*TP53* mutation	NA
		F	55	PDAC	NA	NGS DNA[12]	*TNS3 (27)::MET (15)*	NA	WT	*GNAS* mutation	NA
		NA	NA	PDAC	NA	NGS DNA[12]	*TNS3::MET*	NA	WT	None	NA
Jonna et al.[24]	623 (3)	NA	NA	PDAC	NA	NGS DNA[13]/RNA[14]	*ATP1B1 (2)::NRG1 (1)*	NA	WT	None	NA
		NA	NA	PDAC	NA	NGS DNA[13]/RNA[14]	*CDH1 (3)::NRG1 (2)*	NA	WT	None	NA
		NA	NA	PDAC	NA	NGS DNA[13]/RNA[14]	*VTCN1 (2)::NRG1 (4)*	NA	WT	None	NA
Jones et al.[25]	47 (3)	M	54	PDAC	Head	WGTA	*APP (15-16):: NRG1 (6-7)*	Afatinib	WT	None	Significant response for 5 months (Alive)

		M	59	PDAC	NA	WGTA	*ATP1B1 (3)::NRG1 (2)*	Afatinib	WT	*GNAS* amp *MYC* amp	Significant response at 3 months. PD after 5.5 months (Alive)
		M	55	PDAC	NA	WGTA	*ATP1B1 (4)::NRG1 (6)*	Not treated	WT	*MYC* amp	NA
McEvoy et al.[26]	1 (1)	M	33	PACC	Tail	NGS DNA[15], RT-PCR, Sanger sequencing	*GATM (1-2)::RAF1 (10-17)*	Trametinib	WT	*CDKN2A/B* HD	PD after 1 month (DOD)
Chou et al.[27]	40 (8)	M	64	PACC	NA	NGS RNA[16], FISH	*RET::CCDC6*	NA	NA	NA	NA
		M	55	PACC	NA	NGS RNA[16], FISH	*RET* rearrangement *NOS*	NA	NA	NA	NA
		M	67	PACC	NA	NGS RNA[16], FISH	*BRAF* rearrangement *NOS*	NA	NA	NA	NA
		M	63	PACC	NA	NGS RNA[16], FISH	*BRAF* rearrangement *NOS*	NA	NA	NA	NA
		F	76	PACC	NA	NGS RNA[16], FISH	*BRAF* rearrangement *NOS*	NA	NA	NA	NA

		M	59	PACC	NA	NGS RNA[16], FISH	*BRAF* rearrangement *NOS*	NA	NA	NA	NA
		M	34	PACC	NA	NGS RNA[16], FISH	*BRAF* rearrangement *NOS*	NA	NA	NA	NA
		M	55	PACC	NA	NGS RNA[16], FISH	*RET* rearrangement *NOS*	NA	NA	NA	NA
Singhi et al.[28]	20 (20)	M (6)<br>F (4)	60.5 (43-75)	IOPN/PDAC	Head (5)<br>Body (3)<br>Tail (2)	NGS RNA[17], FISH	10 x *ATP1B1 (1)::PRKACB (2-10)*	NA	WT	None	NA
		M (3)<br>F (1)	57.5 (47-72)	IOPN/PDAC	Head (3)<br>Tail (1)	NGS RNA[17], FISH	4 x *ATP1B1 (1)::PRKACA (2-10)*	NA	WT	None	NA
		M (5)<br>F (1)	53.5 (25-74)	IOPN/PDAC	Head (1)<br>Body (3)<br>Head & Body (1)<br>Tail (1)	NGS RNA[17], FISH	6 x *DNAJB1 (1)::PRKACA (2-10)*	NA	WT	None	NA
Vyas et al.[29]	5 (5)	M	63	IOPN	Head	MSK Solid Tumor Fusion Panel[18]	*ATP1B1::PRKACA*	NA	WT	None	NA
		M	36	IOPN	Head	MSK-IMPACT[6], FISH	*DNAJB1::PRKACA*	NA	WT	None	NA

		F	58	IOPN	Head	MSK-IMPACT[6]	*DNAJB1::PRKACA*	NA	WT	*BRAF* V600_K601delinsE	NA
		M	52	IOPN	Head	MSK-IMPACT[6], FISH	*DNAJB1::PRKACA*	NA	G12D	None	NA
		M	76	IOPN	NA	MSK-IMPACT[6]	*DNAJB1::PRKACA*	NA	G12R	*ERBB2* mutation	NA
O'Reilly et al.[30]	1 (1)	F	61	PDAC	Body	MSK-IMPACT[6]/ NGS RNA[14]	*CTRC (1)::NTRK1 (8)*	Larotrectinib /Selitrectinib	WT	*CDKN2A/B* HD *SMAD4* HD	PR at 2 months. PD after 6 months DOD after 4 months
Lee et al.[31]	9 (1)	F	60	PDAC	NA	NGS RNA[19]	*IQSEC1*::*RAF1*	NA	G12D	None	NA
Hong et al.[32]	2 (2)	NA	NA	PC *NOS*	NA	NGS, FISH, RT-PCR	*NTRK* fusion *NOS*	Larotrectinib	NA	NA	PR for 3.5 months
		NA	NA	PC *NOS*	NA	NGS, FISH, RT-PCR	*NTRK* fusion *NOS*	Larotrectinib	NA	NA	NS
Berger et al.[33]	4 (2)	M	19	PB	NA	Transcriptome sequencing	*EWSR1::FLI1*	NA	WT	None	NA

		F	20	PB	Tail	NGS RNA[14]	*FGFR2::INA*	Nintedanib	WT	None	Treatment was interrupted after 1 month. Patient DOD 1 month later.
Prall et al.[34]	29 (4[d])	M	79	PACC	NA	Targeted DNA[15]/RNA sequencing, FISH, RT-PCR, Sanger sequencing	*GOLGA4::RAF1*	NA	WT	*CDKN2B* HD *SMAD4* HD	NA
		M	79	PACC	NA	Targeted DNA[15]/RNA sequencing, FISH, RT-PCR, Sanger sequencing	*PDZRN3::RAF1*	NA	WT	None	NA
		M	53	PACC-NEC	NA	Targeted DNA[15]/RNA sequencing, FISH, RT-PCR, Sanger sequencing	*HERPUD1::RAF1*	NA	WT	*CDKN2A* HD	NA
		F	29	PACC	NA	Targeted DNA[15]/RNA sequencing, FISH, RT-PCR, Sanger sequencing	*TRIM33::RAF1*	NA	WT	*SMAD4* mutation	NA
Gower et al.[35]	1 (1)	F	41	PDAC	NA	Molecular analysis *NOS*, IHC	*PPFIBP1::ALK*	Alectinib/ Lorlatinib	WT	None	PR for 2 months. Then, PD after 5 months. Eventually SD with the alternative ALKi (Alive)

Gupta et al.[36]	1 (1)	M	81	PACC	Body	FoundationOne CDx[20]	*SEL1L::NTRK1*	Larotrectinib	WT	None	Exceptional Response for >13 months (Alive)
Fusco et al.[37]	100 (4)	M	81	PDAC	Head	Moffitt STAR[21]	*PDZRN3::RAF1*	Trametinib	WT	None	PR on primary tumor but PD on liver met at 3 weeks
		F	56	PDAC	NA	Moffitt STAR[21]	*ATP1B1::NRG1*	Afatinib	WT	None	SD for 4 months. Then, PD (Alive)
		M	80	PDAC	NA	Moffitt STAR[21]	*RDX::MET*	Crizotinib	WT	None	CR for >12 months (Alive)
		F	48	PDAC	Head	FoundationOne CDx[20]	*FGFR2* rearrangement (Intron 17)	Erdafitinib	WT	None	PR for 2 months (Alive)
Melisi et al.[38]	58 (2)	F	48	PACC	NA	FoundationOne CDx[20]	*KANK4::RAF1*	Sorafenib/ Regorafenib	WT	*CDKN2A/B* HD *SMAD4* mutation	PR for 8 months then PD. SD for 4 months then PD.
		NA	NA	Pancreatic mixed adenoneuroendocrine carcinoma	NA	FoundationOne CDx[20]	*TRIM44::BRAF*	NA	WT	*TP53* mutation	NA
Hendifar et al.[39]	3781 (25)	F	52	PDAC	NA	FoundationOne[3]	*MBNL2::BRAF*	Trametinib	WT	None	PR for 18$^{1/4}$ months. Then, PD

		F	57	PACC	NA	FoundationOne[3]	*KANK4::RAF1*	Trametinib	WT	None	PR for > $5^{1/4}$ months
		M	61	PACC	NA	FoundationOne[3]	*SND1::BRAF*	Trametinib	WT	None	SD for $7^{1/2}$ months. Then, PD
		M	52	PACC (Mixed Ductal)	NA	FoundationOne[3]	*SND1::BRAF*	Trametinib	WT	*SMAD4* mutation	SD for 3 months. Then, PD
		F	70	PDAC	NA	FoundationOne[3]	*LUC7L2::BRAF*	pan-RAF inhibitor	WT	*TP53* mutation	PD after 2 months
		F	57	PDAC	NA	FoundationOne[3]	*SND1::BRAF*	Dabrafenib+ Trametinib	WT	*CDKN2A/B* HD	Terminated due to intolerance
		M	56	PDAC	NA	FoundationOne[3]	*SND1::BRAF*	NA	WT	None	NA
		F	38	PDAC	NA	FoundationOne[3]	*EPS15L1::BRAF*	NA	WT	None	NA
		M	70	PACC (Mixed Neuroendocrine)	NA	FoundationOne[3]	*GIPC2::BRAF*	NA	WT	None	NA

		M	80	PACC	NA	FoundationOne[3]	*GLI2::BRAF*	NA	WT	None	NA
		M	86	PDAC	NA	FoundationOne[3]	*JHDM1D::BRAF*	NA	WT	*TP53* mutation<br>CDKN2A *mutation*<br>SMAD4 *mutation*	NA
		M	75	PDAC	NA	FoundationOne[3]	*MKRN1::BRAF*	NA	WT	*TP53* mutation<br>*CDKN2A/B* HD<br>SMAD4 *mutation*	NA
		M	61	PDAC	NA	FoundationOne[3]	*MYRIP::BRAF*	NA	WT	None	NA
		M	63	PDAC	NA	FoundationOne[3]	*RAF1::JAK1*	NA	WT	*CDKN2A/B* HD	NA
		M	59	PDAC	NA	FoundationOne[3]	*SND1::BRAF*	NA	WT	None	NA
		F	56	PDAC	NA	FoundationOne[3]	*SND1::BRAF*	NA	WT	None	NA
		F	67	PACC (Mixed Neuroendocrine)	NA	FoundationOne[3]	*SND1::BRAF*	NA	WT	None	NA

		M	71	PACC	NA	FoundationOne[3]	*SND1::BRAF*	NA	WT	*CDKN2A/B* HD	NA
		M	66	PDAC	NA	FoundationOne[3]	*SND1::BRAF*	NA	WT	*SMAD4* mutation	NA
		F	45	PACC (Mixed Neuroendocrine)	NA	FoundationOne[3]	*SND1::BRAF*	NA	WT	None	NA
		M	46	PACC (Mixed Neuroendocrine)	NA	FoundationOne[3]	*SND1::BRAF*	NA	WT	None	NA
		F	51	PACC (Mixed Ductal)	NA	FoundationOne[3]	*SND1::BRAF*	NA	WT	*CDKN2A/B* HD	NA
		F	57	PDAC	NA	FoundationOne[3]	*STRN3::BRAF*	NA	WT	None	NA
		M	72	PDAC	NA	FoundationOne[3]	*SVOPL::BRAF*	NA	WT	None	NA
		F	42	PDAC	NA	FoundationOne[3]	*TMEM9::BRAF*	NA	WT	*TP53* mutation<br>*CDKN2A/B* HD	NA

Ou et al.[40]	1 (1)	M	34	PDAC	Body, Tail	NGS DNA[22], FISH, IHC	*EML4 (6)::ALK (20)*	Crizotinib/ Alectinib	WT	None	SD for 20 months (Alive)
Poon et al.[41]	1 (1)	F	68	PDAC	Head	FoundationOne CDx[20]	*FGFR2::TACC 2*	Erdafitinib	WT	None	CR for 10 months (Alive)
Allen et al.[42]	400 (3)	M	80-85	PDAC	NA	WGS, RNA sequencing	*EML4 (6)::NTRK3 (14)*	NA	WT	*TP53* mutation *CDKN2A* HD	NA
		M	55-60	PDAC	NA	WGS RNA, sequencing	*EML4 (6)::NTRK3 (14)*	NA	WT	*CDKN2A* HD	NA
		F	70-75	PDAC	NA	WGS, RNA sequencing	*KANK1 (3):: NTRK3 (14)*	NA	G12R[e]	*GNAS* mutation *TP53* mutation	NA
Ma et al.[43]	1 (1)	F	60	PDAC	Head/Neck	NGS DNA[23], Sanger sequencing	*ERC1(1-16)::RET (12-20)*	NA	WT	*TP53* mutation *PIK3CA* mutation *CDKN2A/B* HD	NA
Ghosh et al.[44]	36 (6)	M (3) F (3)	71 (63-78)	PACC (5) PACC-NEC (1)	Head (3) Body (1) Body/Tail (1) Tail (1)	FISH	6 x *BRAF* rearrangement *NOS*	NA	NA	NA	NA
Wang et al.[45]	1 (1)	F	7	PB	Head	NGS DNA[24]	*EML4 (16)::ALK (2)*[25] *EML4 (5)::ALK (2)*[26] *ROS1 (32)::SLC34A2 (7)*[27]	NA	WT	*PTEN* mutation *PIK3CA* mutation	NA

Gaule et al.[46]	1 (1)	M	62	PACC	Head	FoundationOne CDx[20], FISH, IHC	*KANK4*::*ALK*	Alectinib	WT	None	PR for 4 months (Alive)
Schram et al.[47]	2 (2)	M	50	PDAC	NA	MSK Solid Tumor Fusion Panel[18]	*ATP1B1 (2)::NRG1 (2)*	Zenocutuzumab	WT	None	PR for 14 months. Then, PD
		M	34	PDAC	NA	MSK Solid Tumor Fusion Panel[18]	*ATP1B1 (2)::NRG1 (2)*	Zenocutuzumab	WT	None	SD for 11 months. Then, PD
Demetri et al.[48]	4 (4)	NA	NA	PC *NOS*	NA	NGS, PCR	*NTRK* fusion *NOS*	Entrectinib	NA	NA	Response *NOS* for 12.9 months (median duration of response)
		NA	NA	PC *NOS*	NA	NGS, PCR	*NTRK* fusion *NOS*	Entrectinib	NA	NA	Response *NOS* for 12.9 months (median duration of response)
		NA	NA	PC *NOS*	NA	NGS, PCR	*NTRK* fusion *NOS*	Entrectinib	NA	NA	Response *NOS* for 12.9 months (median duration of response)
		NA	NA	PC *NOS*	NA	NGS, PCR	*NTRK* fusion *NOS*	Entrectinib	NA	NA	NS
Subbiah et al.[49]	4 (1)	NA	NA	PC *NOS*	NA	Locally available techniques, FoundationOne[3]	*FGFR2-USP33*	Pemigatinib	WT	*CDKN2A/B* HD	PR for 10.7 months, then PD (Alive)

Philip et al.[50]	2483 (50)	NA	NA	PDAC	NA	NGS DNA[13]/RNA	15 x *BRAF* fusion *NOS*	NA	WT	NA	NA
		NA	NA	PDAC	NA	NGS DNA[13]/RNA	12 x *FGFR2* fusion *NOS*	NA	WT	NA	NA
		NA	NA	PDAC	NA	NGS DNA[13]/RNA	6 x *ALK* fusion *NOS*	NA	WT	NA	NA
		NA	NA	PDAC	NA	NGS DNA[13]/RNA	3 x *RET* fusion *NOS*	NA	WT	NA	NA
		NA	NA	PDAC	NA	NGS DNA[13]/RNA	3 x *NRG1* fusion *NOS*	NA	WT	NA	NA
		NA	NA	PDAC	NA	NGS DNA[13]/RNA	3 x *RAF1* fusion *NOS*	NA	WT	NA	NA
		NA	NA	PDAC	NA	NGS DNA[13]/RNA	2 x *MET* fusion *NOS*	NA	WT	NA	NA
		NA	NA	PDAC	NA	NGS DNA[13]/RNA	2 x *ARHGAP26* fusion *NOS*	NA	WT	NA	NA

		NA	NA	PDAC	NA	NGS DNA[13]/RNA	2 x *ROS1* fusion *NOS*	NA	WT	NA	NA
		NA	NA	PDAC	NA	NGS DNA[13]/RNA	2 x *Fusion NOS*[f]	NA	WT	NA	NA
Ambrosini et al.[51]	5 (5)	M	75	PDAC	NA	NGS DNA	*ALK* rearrangement (Intron 19)	Alectinib	WT	None	PD after 1 month
		M	34	PDAC	NA	NGS DNA	*STRN::ALK*	Crizotinib/ Alectinib	WT	None	PR for 28.4 months
		F	41	PDAC	NA	NGS DNA, IHC	*PPFIBP1::ALK*	Alectinib/ Lorlatinib	WT	*CDKN2A/B* HD	PR for 5 months
		M	63	PDAC	NA	NGS DNA	*EML4::ALK*	Alectinib	WT	*TP53* mutation	SD for 5.4 months
		M	72	PDAC	NA	NGS DNA	*EML4::ALK*	Alectinib	WT	*TP53* mutation *SMAD4* mutation	NA
Paoli et al.[52]	1 (1)	M	6	PACC	NA	NGS RNA[28]	*AGAP3 (9)::BRAF (9)*	NA	WT	None	NA

Subbiah et al.[53]	4 (4)	M	Early 30s	PC *NOS*	Head	NGS or FISH or GeneTrails Solid Tumor Fusion Panel[29]	*RET::TRIM33* *RET::JMJD1C*	Pralsetinib	WT	None	CR for 33.1 months (Alive)
		NA	NA	PC *NOS*	NA	NGS or FISH or GeneTrails Solid Tumor Fusion Panel[29]	*RET::CCDC6*	Pralsetinib	WT	None	PR for 8 months
		NA	NA	PC *NOS*	NA	NGS or FISH or GeneTrails Solid Tumor Fusion Panel[29]	*RET::CCDC6*	Pralsetinib	WT	None	PR for 6 months
		NA	NA	PC *NOS*	NA	NGS or FISH or GeneTrails Solid Tumor Fusion Panel[29]	*RET::CCDC6*	Pralsetinib	WT	None	PR for 6 months
Mafficini et al.[54]	16 (5)	M	76	ITPN/PDAC	Head/Body/Tail	NGS DNA[30]	*FGFR2 (17)::HSD17B4 (13)*	NA	WT	None	NA
		M	68	ITPN/PDAC	Head	NGS DNA[30]	*RET (12)::C14orf93 (3)*	NA	WT	*EP300* mutation	NA
		F	75	ITPN/PDAC	Head/Body/Tail	NGS DNA[30]	*TRIM24 (9)::RET (12)*	NA	WT	*NTRK1* amp	NA
		F	64	ITPN/PDAC	Body/Tail	NGS DNA[30]	*FGFR2 (17)::SYCP1 (24)*	NA	WT	None	NA

		F	63	ITPN/PDAC	Head	NGS DNA[30]	*ERBB2 (24)::P3H4 (2)*	NA	G12D	*TP53* mutation *SMAD4* mutation	NA
Subbiah et al.[55]	11 (11)	NA	NA	PDAC	NA	NGS, FISH, PCR	11 x *RET* fusions *NOS*	Selpercatinib	NA	NA	PR for 6 patients
Maimaitiali et al.[56]	14 (14)	M (2)	64.5 (62-67)	IOPN	Head (2)	RT-PCR, DNA sequencing	2 x *ATP1B1::PRKACB*	NA	WT	None	NA
		M (3) F (4)	74 (47-82)	IOPN	Body (4) Body & Tail (3)	RT-PCR, DNA sequencing	7 x *ATP1B1::PRKACA*	NA	WT	None	NA
		M (3) F (2)	69 (44-78)	IOPN	Head (2) Body & Tail (2) Tail (1)	RT-PCR, DNA sequencing	5 x *DNAJB1::PRKACA*	NA	G12D (1)	None	NA
Ng et al.[57]	1 (1)	M	28	PDAC	Neck	FoundationOne[3],Perthera[31]	*FGFR2* rearrangement (Intron 17)	Erdafitinib	WT	*CDKN2A/B* HD	Exceptional response for >12 months (Alive)
Paniccia et al.[58]	192 (4)	NA	NA	IPMN/ PDAC	NA	NGS DNA/RNA[32]	*SND1 (9)::BRAF (9)*	NA	WT	*TP53* mutation *GNAS* mutation	NA
		NA	NA	IPMN/ PDAC	NA	NGS DNA/RNA[32]	*SND1 (9)::BRAF (9)*	NA	WT	None	NA

		NA	NA	IPMN/ PDAC	NA	NGS DNA/RNA[32]	*TRIM24(3)::BRAF (10)*	NA	WT	*GNAS* mutation *PIK3CA* mutation	NA
		NA	NA	IPMN/ PDAC	NA	NGS DNA/RNA[32]	*AGK (2)::BRAF (8)*	NA	WT	*GNAS* mutation *TP53* mutation	NA
Topham et al.[59]	63 (6)	NA	NA	PDAC	NA	WGS	*NRG1::ATP1B1*	NA	WT	None	NA
		NA	NA	PDAC	NA	WGS	*NRG1::ATP1B1*	NA	WT	None	NA
		NA	NA	PDAC	NA	WGS	*NRG1::APP*	NA	WT	None	NA
		NA	NA	PDAC	NA	WGS	*FGFR2::GCC2*	NA	WT	*SMAD4* HD	NA
		NA	NA	PDAC	NA	WGS	*NTRK2::THAP1*	NA	WT	None	NA
		NA	NA	PDAC	NA	WGS, RNA sequencing	*BRAF::TNS3*	NA	WT	*SMAD4* mutation *CDKN2A* HD	NA

Helal et al.[60]	4 (4)	M	82	PDAC	Head	FoundationOne CDx[20]	*FGFR2::CCSER2*	FGFR2 inhibitor	WT	*CDKN2A/B* HD *NRAS* amp *NTRK1* amp	Under treatment for 6 months, NOS. (Alive)
		M	79	PDAC	Head	FoundationOne CDx[20]	*FGFR2::CGNL1*	FGFR2 inhibitor	WT	None	Under treatment for 10 months, NOS. (Alive)
		F	62	PDAC	Head	FoundationOne CDx[20]	*FGFR2-KCTD1*	FGFR2 inhibitor	WT	None	Under treatment for 8 months, NOS. (Alive)
		M	72	PDAC	Body	FoundationOne CDx[20]	*FGFR2-INA*	FGFR2 inhibitor	WT	None	Under treatment for 2 months, NOS. (Alive)
Thavaneswaran et al.[61]	1 (1)	F	38	PDAC	NA	NGS DNA/RNA	*ATP1B1 (2)::NRG1 (2)*	Seribantumab	WT	*JUN* mutation	PR for 6 months (Alive)
Goto et al.[62]	1 (1)	M	10	PB	NA	FoundationOne CDx[20]	*IQSEC1 (12)::RAF1 (10)*	Dabrafenib+ Trametinib	WT	*MAP3K1* mutation	SD for 4 months (DOD)
Agaimy et al.[63]	4 (4)	M	53	ACTH-producing pNEN	NA	NGS RNA[33], FISH	*EWSR1::BEND2*	NA	WT	None	NA
		F	36	ACTH-producing pNEN	NA	NGS RNA[33], FISH	*EWSR1::BEND2*	NA	WT	None	NA

		M	29	ACTH-producing pNEN	NA	NGS RNA[33]	*KMT2A::BCOR*	NA	WT	*TSC1* mutation	NA
		F	48	ACTH-producing pNEN	NA	NGS RNA[33]	*TFG::ADGRG7*	NA	WT	None	NA
Lee et al.[64]	23 (19)	M	34	ITPN/PDAC	Head	NGS DNA/RNA[32], WTS	*STRN::ALK*	NA	WT	*TP53* HD *SMAD4* HD	NA
		M	62	ITPN/PDAC	Head	NGS DNA/RNA[32], WTS	*SND1::BRAF*	NA	WT	*TP53* HD	NA
		F	18	ITPN/PDAC	Entire pancreas	NGS DNA/RNA[32], WTS	*AGK::BRAF*	NA	WT	*TP53* HD *SMAD4* HD	NA
		M	59	ITPN/PDAC	Body/Tail	NGS DNA/RNA[32], WTS	*ATP1B1::NRG1*	NA	WT	*SMAD4* HD	NA
		M	79	ITPN/PDAC	Entire pancreas	NGS DNA/RNA[32], WTS	*ATP1B1::NRG1*	NA	WT	*SMAD4* HD	NA
		F	68	ITPN	Body/Tail	NGS DNA/RNA[32], WTS	*TRIM24::RET*	NA	WT	*CDK2NA/B* HD *SMAD4* HD	NA

		F	61	ITPN	Head	NGS DNA/RNA[32], WTS	*FGFR2::BICC1*	NA	WT	*CDKN2A/B* HD	NA
		F	27	ITPN/PDAC	Head	NGS DNA/RNA[32], WTS	*FGFR2::CIT*	NA	WT	*CDKN2A/B* HD	NA
		F	81	ITPN/PDAC	Body	NGS DNA/RNA[32], WTS	*FGFR2::TACC2*	NA	WT	*TP53* mutation *MYC* amp	NA
		F	78	ITPN/PDAC	Tail	NGS DNA/RNA[32], WTS	*FGFR2::TACC2*	NA	WT	*SMAD4* HD	NA
		M	73	ITPN/PDAC	Head	NGS DNA/RNA[32], WTS	*FGFR2::FAM76A*	NA	WT	*TSC1* mutation	NA
		M	63	ITPN/PDAC	Body/Tail	NGS DNA/RNA[32], WTS	*FGFR2::PPHLN1*	NA	WT	*MYC* amp	NA
		M	22	ITPN	Entire pancreas	NGS DNA/RNA[32], WTS	*FGFR2::NOL4*	NA	WT	*CDKN2A/B* HD	NA
		F	53	ITPN/PDAC	Entire pancreas	NGS DNA/RNA[32], WTS	*CFTR::RASGRF2*	NA	WT	*MYC* amp	NA

		F	37	ITPN/PDAC	Head	NGS DNA/RNA[32], WTS	*SPTLC1::JAK2*	NA	WT	*SMAD4* HD	NA
		M	74	ITPN/PDAC	Entire pancreas	NGS DNA/RNA[32], WTS	*ATXN3::MAML3*	NA	WT	*TP53* mutation *PTEN* mutation *CDKN2A/B* HD	NA
		F	68	ITPN/PDAC	Head	NGS DNA/RNA[32], WTS, FISH	*NCOA3::MAML2*	NA	WT	*TP53* mutation *SMAD4* HD *CDKN2A/B* HD	NA
		F	73	ITPN	Head	NGS DNA/RNA[32], WTS, FISH	*RUNX2::MAML2*	NA	WT	*BRCA2* mutation *TP53* HD *CDKN2A/B* HD	NA
		M	47	ITPN/PDAC	Head	NGS DNA/RNA[32], WTS, FISH	*ATXN3::MAML2*	NA	WT	None	NA
Zhao et al.[65]	499 (1)	NA	NA	PDAC	NA	NGS DNA[34]	*NCOA4::RET*	NA	WT	None	NA
Zhang et al.[66]	1 (1)	M	68	PDAC	NA	NGS DNA[35]	*TRIM33::RET*	Pralsetinib	WT	*TGFBR1* mutation	PR for >12 months (Alive)
Reutter et al.[67]	1 (1)	F	55	PDAC	NA	NGS DNA[36]/RNA sequencing[36], FISH, IHC	*SLC4A4 (1-21)::ROS1 (34-43)*	Crizotinib/ Lorlatinib	WT	*FGFR4* mutation	PR for 12 months. Then PD. Then PR for 12 months with the alternative ALKi (Alive)

Reissig et al.[68]	190 (5)	NA	NA	PC *NOS*	NA	NGS RNA[37]	*NOS1AP::NTRK1*	Larotrectinib	WT	NA	Exceptional Response for 15 months (Alive)
		NA	NA	PC *NOS*	NA	NGS RNA[37]	*TNS3::BRAF*	Regorafenib/ Trametinib	WT	NA	NA[g]
		NA	NA	PC *NOS*	NA	NGS RNA[37]	*FGFR2::CREB5*	Not treated	WT	NA	NA[h]
		NA	NA	PC *NOS*	NA	NGS RNA[37]	*ERC1::RET*	Not treated	WT	NA	NA[h]
		NA	NA	PC *NOS*	NA	NGS RNA[37]	*PDZRN3::RAF1*	Not treated	WT	NA	NA[h]
Severson et al.[69]	157 (1)	F	41	PC *NOS*	NA	NGS RNA[38]	*SLC3A2 (5)::NRG1 (2)*	NA	WT	*SMAD4* mutation	NA
He et al.[70]	47 (2)	NA	NA	PDAC	NA	WES	*BRCA1 (1-11)::RPRML (3-19)*	NA	NA	NA	NA
		NA	NA	PDAC	NA	WES	MIR943 (Intergenic)*::FGFR3 (3-19)*	NA	NA	NA	NA

Simbolo et al.[71]	20 (1)	M	58	PDAC	Head/Body	NGS DNA[30]	*RET (12)::CCDC6 (8)*	NA	WT	*APC* mutation	NA
Heinrich et al.[72]	1 (1)	NA	NA	PDAC	NA	NGS RNA[32, 39]	*TRIM24 (10)::BRAF (9) BRAF (8)::TRIM24 (11)*	NA[40]	WT	NA	NA
Sun et al.[73]	7 (1)	M	66	PACC (Mixed Neuroendocrine)	Body	NGS DNA[32]	*RET::CCDC6*	NA	WT	None	NA

Notes:

[1]NGS-based DNA sequencing analyzed all coding exons of 236 or 405 cancer-related genes and 47 introns of 19 genes frequently rearranged in cancer; [2]NGS-based RNA sequencing analyzed rearrangements involving 265 genes (please see Supplementary Tables S1 and S2 of the ***reference 13***); [3]NGS-based DNA sequencing utilizing a panel of 287 cancer-related genes; [4]Targeted NGS of 295 genes and gene fusion detection of 19 genomic rearrangements (online reference: https://jnccn.org/supplemental/journals/jnccn/15/5/article-p555.xml/jnccn01505555_Supplemental_material.pdf); [5]Colloid carcinoma is a rare histopathological subtype of PDAC; [6]NGS-based DNA sequencing utilizing a panel of 341 cancer-related genes (please see supplementary material of the ***reference 18***); [7]NGS-based DNA sequencing utilizing a panel of 409 cancer-related genes (please see supplementary material of the ***reference 18***); [8]NGS-based DNA sequencing utilizing a panel of 35 cancer-related genes previously reported to be involved in chromosomal rearrangements; [9]This study included 31 cases previously investigated (Chmielecki et al., previous cohort) and new 18 cases (new cohort). In the new cohort, the investigators detected 4 cases with fusion genes, but here we added another one belonging to the previous cohort, not detected in the study by Chmielecki et al. with DNA sequencing, but detected with FISH in the current study (Wang et al.); [10]TrustSeq RNA sample preparation kit v2 (online reference: https://emea.illumina.com/products/by-type/sequencing-kits/library-prep-kits/truseq-rna-v2.html); [11]Trailblaze Pharos™ is a multiplex NGS-based assay for the qualitative detection of fusions in the NTRK1/2/3, ROS1, or ALK genes in the RNA from formalin-fixed paraffin-embedded (FFPE) human solid tumor tissue; [12]A panel of 315 cancer-related genes and selected introns from 28 genes frequently rearranged in cancer (please see supplementary material of the ***reference 23***); [13]A custom-designed SureSelect XT assay of 592 whole-gene targets; [14]Archer FUSIONPlex Core Solid Tumor Panel, NGS-based RNA sequencing utilizing a panel of 57 cancer-related genes; [15]NGS-based DNA sequencing utilizing a panel of 386 cancer-related genes; [16]In this study, a custom designed Ion AmpliSeq RNA panel and Trusight RNA fusion panel were used; [17]The OCAv3 RNA panel evaluates 760 fusion genes that comprise intergenic and intragenic regions among 51 cancer-related kinase genes (please see supplementary material of the ***reference 28***); [18]RNA-based panel that utilizes PCR and NGS technology to detect gene fusions in 62 genes; [19]Archer FusionPlex involving 36-53 cancer-related genes; [20]NGS-based DNA sequencing utilizing a panel of 309 cancer-related genes (online reference: https://www.rochefoundationmedicine.com/f1cdxtech); [21]NGS-based DNA and RNA sequencing utilizing a panel (TruSight Tumor 170) of 170 and 55 cancer-related genes, respectively; [22]NGS-based DNA sequencing utilizing a panel of 520 cancer-related genes; [23]ChosenOne599, NGS-based DNA sequencing utilizing a panel of 599 cancer-related genes; [24]NGS-based DNA sequencing utilizing a panel of 704 cancer-related genes; [25]The fusion was detected in the plasma; [26]The fusion was detected in the adjacent tissue; [27]The fusion was detected in the tumor tissue; [28]Archer FusionPlex Comprehensive Sarcoma Panel involving 52 genes involved in translocation-related sarcomas; [29]NGS-based RNA sequencing utilizing a panel of 24 cancer-related genes previously reported to be involved in clinically informative gene fusions (online reference: https://knightdxlabs.ohsu.edu/home/test-details?id=GeneTrails+Comprehensive+Solid+Tumor+Panel); [30]Glasgow Cancer Core panel spans 1.85 Mb of the

genome and includes 174 genes cancer-related genes; [31]A multi-omics molecular analysis (online reference: https://www.perthera.com/our-technology/); [32]Oncomine Comprehensive Assay v3 covers 161 of the most relevant cancer genes; [33]TruSight RNA Fusion panel; [34]An 831-gene panel (Onco Panscan™, Genetron Health) was utilized to identify somatic alterations and gene fusions; [35]NGS-based DNA sequencing utilizing a panel of 733 cancer-related genes; [36]NGS-based DNA and RNA sequencing (TruSight Oncology 500 and TruSight Tumor 170); [37]FusionPlex CTL Panel (please see supplementary material of the ***reference 68)***; [38]OmniSeq INSIGHT, NGS-based DNA sequencing of 523 genes and RNA sequencing of 55 genes; [39]Archer FusionPlex Lung panel (Archer Dx) is a custom Archer panel utilizing a panel of 13 cancer-related genes, namely, *ALK*, *BRAF*, *EGFR*, *ERBB2*, *ERBB4*, *FGFR1*, *FGFR2*, *FGFR3*, *KIT*, *MET*, *NTRK1*, *NTRK2*, *NTRK3*, *RET*, and *ROS1*; [40]The patient suffered from PDAC disease with microsatellite instability and was treated with immunotherapeutic agent achieving excellent response; [a]Best response observed from the start of the treatment with the targeted agent for fusion genes; [b]The survival refers to that documented after treatment initiation with the targeted agent for fusion genes; [c]Nab-paclitaxel+5-fluorouracil+folinic acid; [d]The study described five cases, but one of them has been already reported and counted in the table, since it was previously described by another study (McEvoy et al.); [e]Subclonal mutation; [f]These two fusions are not specified further in the text; [g]Specific data regarding patient's response are not available; [h]Targeted therapy was not administrated or response could not be determined

Abbreviations:

PDAC, Pancreatic ductal adenocarcinoma; NGS, Next-generation sequencing; FISH, Fluorescence in situ hybridization; IHC, Immunohistochemistry; WT, Wild-type; CR, Complete response; PR, Partial response; SD, Stable disease; PD, Progressive disease; DOD, Dead of disease; EBRT, External beam radiotherapy; NOS, not otherwise specified; IMRT, Intensity-modulated radiotherapy; NOI, No other information; WGS, Whole genome sequencing; WTS, Whole transcriptome sequencing; NS, not specified; PC, Pancreatic cancer; IOPN, Intraductal oncocytic papillary neoplasm; ITPN, Intraductal tubulopapillary neoplasm; PACC, Pancreatic acinar cell carcinoma; PB, Pancreatoblastoma; ACTH, Adrenocorticotrophic hormone; HD, Homozygous deletion

Targeted agents' information:

ALK inhibitor: *Ceritinib, Alectinib, Crizotinib, Lorlatinib*; FGFR1-3 inhibitor: *Pemigatinib*; FGFR1-4 kinase inhibitor: *Erdafitinib*; NTRK inhibitor: *Larotrectinib, Selitrectinib*; HER kinase inhibitor: *Afatinib*; Anti-HER2 antibodies: trastuzumab, pertuzumab; Anti-HER2/HER3 bispecific antibody: *Zenocutuzumab*; Anti-HER3 antibody: *Seribantumab*; EGFR TKI: Erlotinib; Tyrosine kinase inhibitor: *Entrectinib, Nintedanib*; MEK inhibitor: *Trametinib*; BRAF inhibitor: Dabrafenib; RET inhibitor: *Selpercatinib, Pralsetinib*; Multikinase inhibitor: *Sorafenib, Regorafenib*.

Supplementary Table 3. Summary of Non-Druggable Gene fusions identified in Pancreatic Tumors.

Main Fusion Gene	*ErbB member*	*EWSR1*	*MAML2/3*	*PRKACA/B*	*Others (single events)**
Partner	*NUMA1* (1)<br>*SEPT14* (1)<br>*P3H4* (1)	*BEND2* (4)<br>*FLI1* (2)	*ATXN3* (2)<br>*NCOA3* (1)<br>*RUNX2* (1)	*ATP1B1* (24)<br>*DNAJB1* (15)	*FGFR3 (17)::TACC3 (11)*<br>*KMT2A::BCOR*<br>*TFG::ADGRG7*<br>*CFTR::RASGRF2*<br>*SPTLC1::JAK2*<br>*BRAC1 (1-11)::RPRML (3-19)*<br>*MIR943 (intergenic)::FGFR3 (3-19)*<br>*ARHGAP26* (2)
Subtype<br>PDAC<br>ITPN/PDAC<br>IOPN/PDAC<br>ITPN<br>IOPN<br>PanNET<br>pNEN<br>PB	 2<br>1<br>-<br>-<br>-<br>-<br>-<br>-	 -<br>-<br>-<br>-<br>-<br>3<br>2<br>1	 -<br>3<br>-<br>1<br>-<br>-<br>-<br>-	 -<br>-<br>20<br>-<br>19<br>-<br>-<br>-	 5<br>2<br>-<br>-<br>-<br>-<br>2<br>-
Gender<br>Male<br>Female	 -<br>3	 4<br>2	 2<br>2	 26<br>13	 2<br>3
Age (Median)	58.0	37.0	70.5	63.0	62.5

Notes: *This table shows all non-actionable fusion genes detected in at least 2 cases, with the last column reporting fusion genes (both partners) detected in only one case (others).

Abbreviations: PDAC: Pancreatic ductal adenocarcinoma; ITPN: Intraductal tubulopapillary neoplasm; IOPN: Intraductal oncocytic papillary neoplasm; PanNET: Pancreatic neuroendocrine tumor; pNEN: Pancreatic neuroendocrine neoplasm; PB: Pancreatoblastoma.

Supplementary Figure 1. PRISMA checklist for this systematic review.

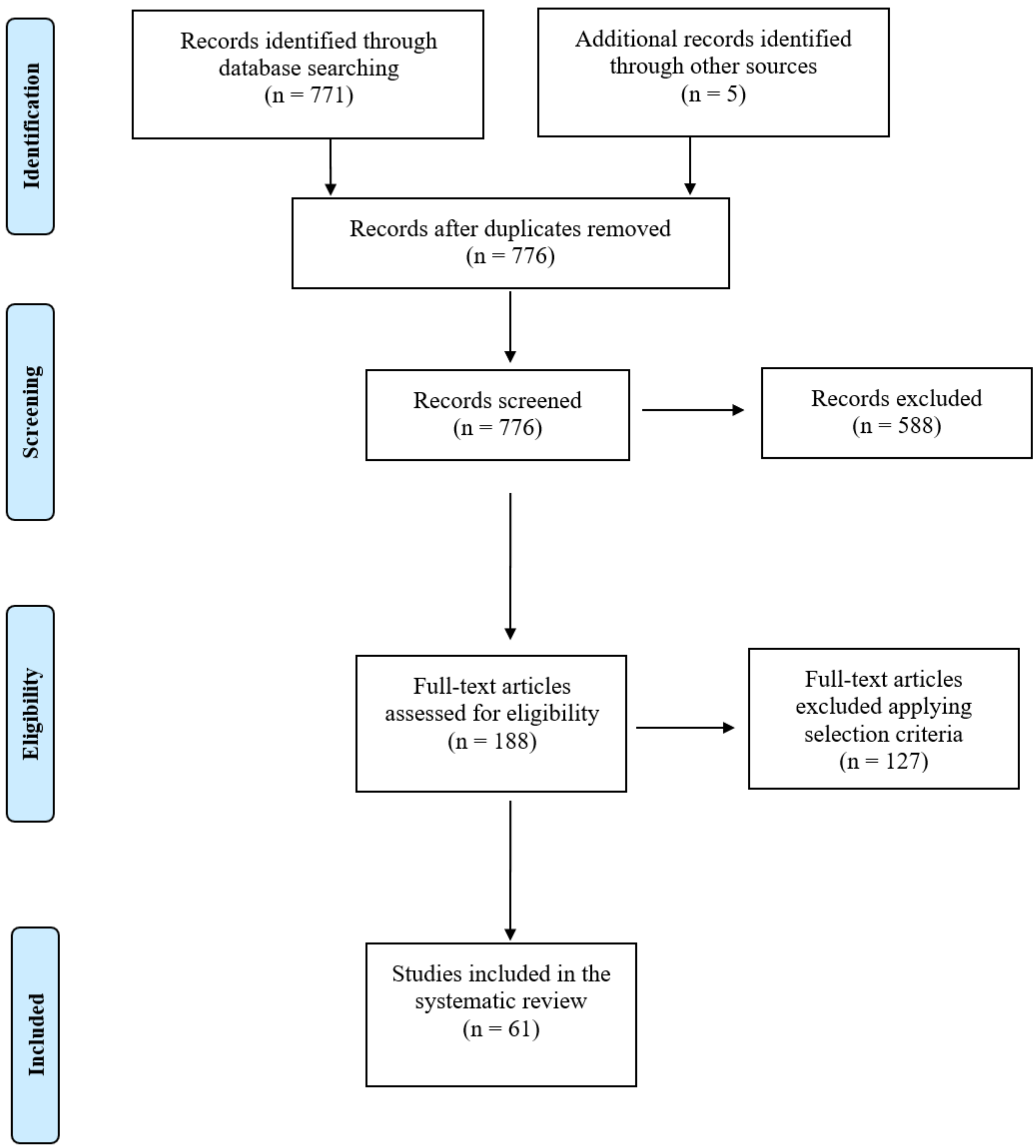

Supplement: Supplementary file 1 [file NIHMS2200879-supplement-Supplementary_Material.pdf]
